# Ventx Factors Function as Nanog-Like Guardians of Developmental Potential in *Xenopus*

**DOI:** 10.1371/journal.pone.0036855

**Published:** 2012-05-14

**Authors:** Pierluigi Scerbo, Fabrice Girardot, Céline Vivien, Gabriel V. Markov, Guillaume Luxardi, Barbara Demeneix, Laurent Kodjabachian, Laurent Coen

**Affiliations:** 1 Département Régulations, Développement et Diversité Moléculaire, Muséum National d’Histoire Naturelle, Paris, France; 2 Institut de Biologie du Développement de Marseille Luminy, Aix-Marseille Université, Marseille, France; 3 WatchFrog S.A., Evry, France; 4 Institut de Génomique Fonctionnelle de Lyon, Université de Lyon, Ecole Normale Supérieure de Lyon, Lyon, France; Radboud University Nijmegen, The Netherlands

## Abstract

Vertebrate development requires progressive commitment of embryonic cells into specific lineages through a continuum of signals that play off differentiation versus multipotency. In mammals, Nanog is a key transcription factor that maintains cellular pluripotency by controlling competence to respond to differentiation cues. *Nanog* orthologs are known in most vertebrates examined to date, but absent from the Anuran amphibian *Xenopus*. Interestingly, *in silico* analyses and literature scanning reveal that basal vertebrate ventral homeobox (ventxs) and mammalian Nanog factors share extensive structural, evolutionary and functional properties. Here, we reassess the role of ventx activity in *Xenopus laevis* embryos and demonstrate that they play an unanticipated role as guardians of high developmental potential during early development. Joint over-expression of *Xenopus ventx1.2* and *ventx2.1-b* (*ventx1/2*) counteracts lineage commitment towards both dorsal and ventral fates and prevents *msx1*-induced ventralization. Furthermore, *ventx1/2* inactivation leads to down-regulation of the multipotency marker *oct91* and to premature differentiation of blastula cells. Finally, supporting the key role of *ventx1/2* in the control of developmental potential during development, mouse *Nanog* (*mNanog*) expression specifically rescues embryonic axis formation in *ventx1/2* deficient embryos. We conclude that during *Xenopus* development ventx1/2 activity, reminiscent of that of Nanog in mammalian embryos, controls the switch of early embryonic cells from uncommitted to committed states.

## Introduction

In vertebrates, early embryonic cells remain undifferentiated prior to gastrulation. As such, they are not restricted to a predetermined fate but can enter a number of differentiation pathways leading to all the cell types of the adult organism. This capacity is referred to as pluripotency. Examples of pluripotent cells include cells of the inner cell mass and epiblast of the mammalian blastocysts [1], [2], [3], [4] and those of the animal pole of *Xenopus* blastulae [5], [6]. During gastrulation the gene network that maintains the undifferentiated state is rewired and embryonic cells gradually lose their initial high developmental potential, which causes lineage restriction and allows the progressive building of organs [7]. In this process, cell fate is tightly controlled by signals that either promote the entry into given differentiation paths, or restrict this capacity and maintain cellular developmental potential.

Studies of pluripotency have uncovered key signals and factors that promote maintenance of the uncommitted state or lineage specification [8], [9], [10]. In mammals, these signals are thought to converge on the POU5F1/SOX2/NANOG triumvirate of transcription factors that constitutes the core network controlling pluripotency [9], [11], [12]. *Nanog*, which encodes a homeodomain-bearing transcription factor of the NKL class, was first identified in mammals as being essential for early embryonic development and germ-line establishment through its capacity to restrain premature differentiation of embryonic stem cells [3], [13], [14], [15]. NANOG activity indeed protects undifferentiated cells against the differentiation-inducing effects of extracellular signals and transcriptional noise [15], [16]. Though *Nanog* was initially thought to be a mammal-specific gene, orthologs have been characterized in most vertebrate species, including birds [17], [18], teleosts [19], [20] and non-anuran (urodele) amphibians [21], [22]. Constitutive expression of a modified axolotl *Nanog* ortholog was shown to sustain pluripotency in mouse ES cells cultured in the absence of LIF [18], [20], [21]; further, functional assays have shown that chick as well as zebrafish *Nanog* orthologs could restore the capacity to reprogram *Nanog*-deficient murine cells to fully pluripotent iPS cells [23], suggesting that this factor controls developmental potential across osteichtyes.In *Xenopus*, uncommitted embryonic cells maintain high developmental potential until the onset of gastrulation [5], [6], similar to *Nanog*-expressing epiblastic cells in amniote embryos [3], [9], [24]. However, much less is known about the molecular mechanism that underlies this cellular property in *Xenopus*. Interestingly, in all tetrapods, including *Xenopus*, uncommitted embryonic cells express transcription factors of the POU5F1 family [18], [21], [25], [26], [27]. In mammals, chick and amphibians, POU5F1s maintain embryonic cells in an uncommitted state, preventing their differentiation [18], [25], [28], [29]. However, if most of the major players in the mammalian pluripotency network are structurally conserved in the *Xenopus* genome [20], [30], so far no *Nanog* ortholog has been identified in anuran amphibians [31]. Thus, either *Nanog* remains to be characterized in anurans or other(s) factor(s) must maintain the high developmental potential of uncommitted embryonic cells in this taxon [5], [6]. Here, we present evidence suggesting that this function is carried out by ventx transcription factors in *Xenopus*.

Members of the VENTX family of NKL transcription factors were first identified in *Xenopus* and owe their name (*vent*ral homeobo*x*) to their ventral marginal zone expression domain in *Xenopus* gastrulae [32], [33]. They form a small multigenic family organized in a compact cluster in most chordate genomes, except mammals where either a single or no *ventx* ortholog is found. *Xenopus* species possess at least 6 *ventx* paralogs, which can be grouped in 3 subclasses: *ventx1s*, *ventx2s* and *ventx3s*. There is longstanding agreement that all *ventx1s* and *ventx2s* function in a similar fashion [34] and the less studied *ventx3s* seem to follow this pattern as well [35], [36]. All ventx factors are known to act as transcriptional repressors and to be expressed in roughly overlapping territories during early and late development, ventx2s being more broadly expressed in spatial and temporal terms [32], [33], [34], [35]. More specifically, they are all expressed in the animal hemisphere of blastulae and the ventral side of early gastrulae, where they participate in the *bmp4*-controlled gene network that acts in the establishment of dorso-ventral patterning [33]. In this “ventral center”, they antagonize dorsalization induced by the Spemann organizer, opposing the spread of the organizer in ventro-lateral domains by regionalizing the expression of organizer-specific genes, such as *gsc* [37]. When overexpressed in *Xenopus* embryos they give rise to ventralized phenotypes, characterized at tailbud stage by anterior truncations, short and/or bent tails and absent or defective axial structures such as notochord and floor plate [32], [33], [34], [38]. Conversely, expression of dominant-negative *ventx* constructs leads to double axis formation [34], whereas *ventxs* knock-down causes severe dorsalization, characterized by the loss of caudal territories and increased neuralization of the ectoderm [37]. Here, we propose a reinterpreted role for ventx factors, as guardians of high developmental potential during early *Xenopus* development. This conclusion is based on the key observation that ventx factors repress differentiation towards dorsal as well as ventral fates and that their knockdown can be rescued by ectopic expression of the mouse pluripotency regulator Nanog. We suggest that this crucial activity protects the future ventral territories from premature commitment towards dorsal fates in order to ensure proper spatio-temporal patterning of the embryo.

## Results

### Ventx and Nanog Factors Share Common Properties

We set out to identify a putative *Nanog* ortholog in *Xenopus*. *In silico* screening of sequence repositories resulted in the detection of annotated or putative *Nanog* orthologs in all gnathostomes, except *Xenopus* species. Degenerate PCR-based approaches were also unsuccessful (**data not shown** and see Supporting Information S1 for Extended Experimental Procedures). Moreover, the synthenic region where *Nanog* orthologs are found in other tetrapods, including axolotl, is conserved in *Xenopus tropicalis* albeit split over two scaffolds in the current state of the genome assembly (see ensembl scaffolds GL173371 and GL173015). These scaffolds contain no *Nanog*-related sequence, strongly arguing that the absence of *Nanog* from the *Xenopus* genus is due to secondary loss. Others have recently reached a similar conclusion [20]. Therefore, we tested the alternative hypothesis that other *Xenopus* transcription factors might be capable of functionally replacing *Nanog*.

As Nanog belongs to the NKL subclass of homeodomain-containing proteins, we focused on this group to identify putative candidates. Phylogenetic reconstruction showed NKL families to be monophyletic, except for NK4 and VENTX (**Fig. S1** ***A***). Surprisingly, the amphioxus Ventx orthologs [39] appear at the base of the NANOG group, suggesting that VENTX and NANOG families might be closely related (**Fig. S1** ***C***). Furthermore, these families share multiple features that are unique among NKLs. Notably, VENTX and NANOG are the only NKL families to have been lost in specific vertebrate lineages: *Nanog* is absent in the *Xenopus* genus whereas, inversely, rodents lack *Ventx*. Also, *VENTX* and *NANOG* are the only NKL to have numerous processed pseudogenes in the human genome (6 and 10 respectively) [40], which often correlates with expression in the germline or its embryonic precursors, and is a proposed signature of genes involved in the maintenance of pluripotency [41]. Finally, VENTX and NANOG have long branches when compared to other NKL families (e.g. NK1 or LBX, see **Fig. S1**), indicating that the homeodomains from these two families are less conserved among vertebrates than those from other NKLs (see also **Table S1**). These shared features make *Xenopus ventxs* good candidates for serving *Nanog*-like functions.

In line with this hypothesis, mammalian *Nanog* and *Xenopus ventx* genes encode transcriptional repressors [34], [42], [43] and share striking functional similarities (summarized in **Table S2**). First, the orthologs of many genes regulated by ventxs in *Xenopus* are regulated by NANOG in mammals; second, *Nanog* and *ventxs* are regulated by the same signalling pathways and transcription factors; third, ventxs and NANOG interact with orthologous proteins. One of the most significant parallels is that, in *Xenopus* and teleosts, endogenous ventx and pou5f1 transcription factors interact physically and genetically during early development [44], [45], [46], as do mammalian NANOG and POU5F1 [9].

### Mouse Nanog and ventx1/2 Overexpression have Similar Effects

**Figure 1:**
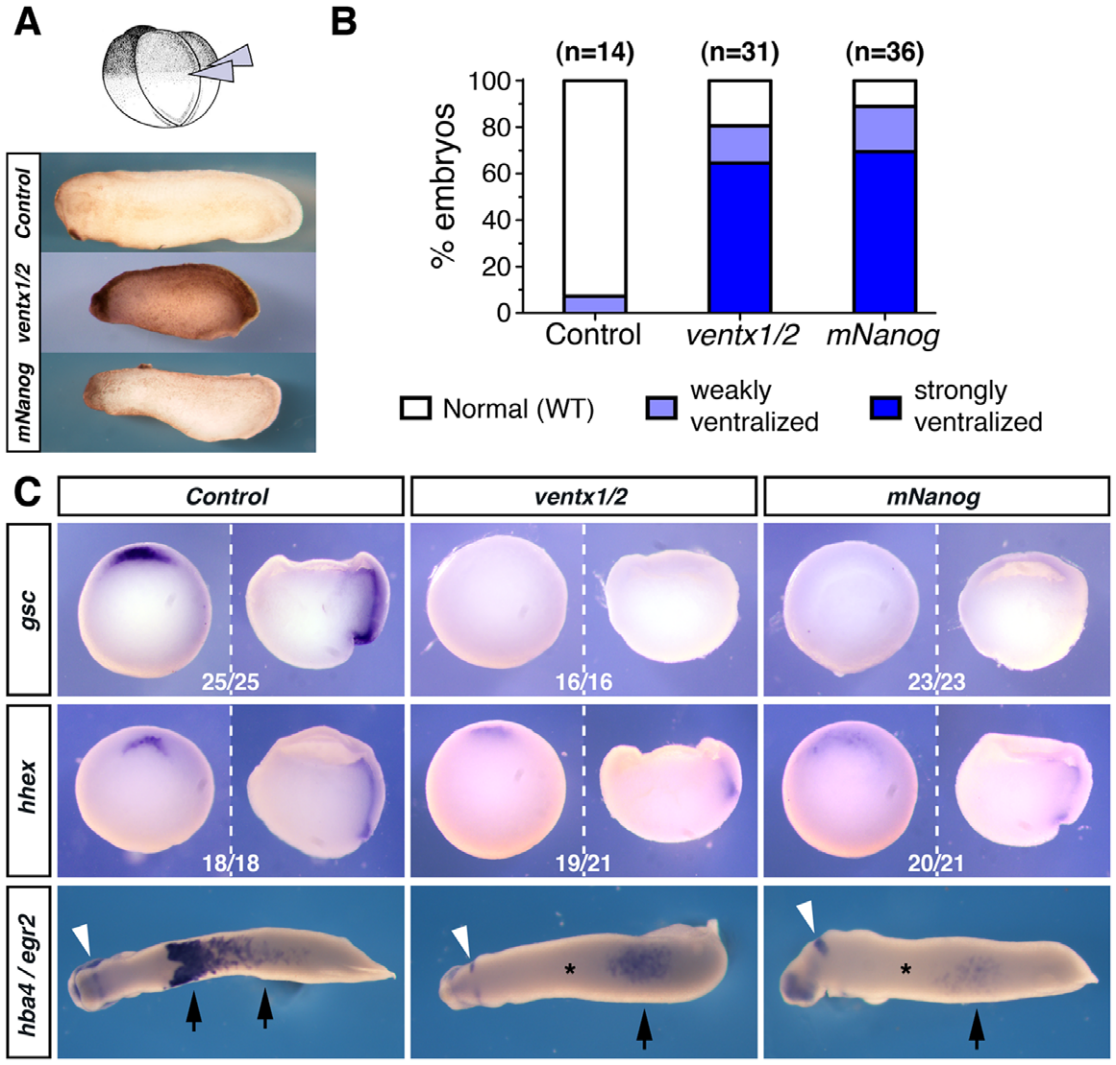
*mNanog* and *ventx1/2* overexpression cause similar effects in *Xenopus* embryos. (***A***) Four-cell stage embryos (NF3) were injected in both dorsal blastomeres, with a 1∶3 mix of *ventx1.2* and *ventx2.1-b* mRNAs (*ventx1/2*; 0.5 ng per blastomere), with mouse *Nanog* mRNA (*mNanog*; 0.15 ng/blastomere), or with water for control. Representative phenotypes observed at tailbud stage (NF28) are shown (lateral views, anterior to the left, dorsal to the top). (***B***) Percentages of observed phenotypes in three independent experiments for mock (n = 14), *ventx1/2* (n = 31) and *mNanog* (n = 36) mRNAs injections. (***C***) Embryos injected as in (***A***) were collected at early gastrulae (NF10.5; whole embryos: ventral view, dorsal side to the top; hemisected embryos: lateral view, dorsal to the left, animal side to the top) and tailbud (NF28; ventral view, anterior to the left) stages and processed for whole-mount *in situ* hybridization (WISH) with a *gsc* or *hhex* probe, or with *hba4* (black arrowheads) and *egr2* (white arrowheads), respectively. The number of embryos showing staining similar to the one photographed over the total number of embryos assayed is indicated.

These extensive similarities prompted us to compare the effects of overexpression of mouse *Nanog* (*mNanog*) to combined *Xenopus ventx1.2* [32] and *ventx2.1-b* [47] (referred to as *ventx1/2* from now on) overexpression on *Xenopus* embryonic development. The relevant mRNAs were dorsally injected at the 4-cell stage (NF3 [48]), using previously described doses for *ventx1/2* (0.5 ng per blastomere [34]) and half the lethal dose for *mNanog* (0.6 ng per blastomere, see **Fig. S2**). As expected [34], *ventx1/2* overexpression led at tailbud stage (NF28) to severely ventralized phenotypes with truncated anterior structures (**Fig. 1** ***A***). Remarkably, *mNanog* overexpression produced similar defects with comparable penetrance (**Fig. 1** **,** ***A*** and ***B*** **,** and **Fig. S3** ***C***). In contrast, overexpression of the medaka ortholog *OlNanog* led to phenotypes clearly distinct from those obtained with *mNanog*, no ventralization being observed at any of the doses assayed, with all embryos displaying clear head features (**Fig. S3A**).

We next checked if *ventx1/2* and *mNanog* dorsal overexpression had similar impacts on gene expression. As previously reported, *ventx1/2* overexpression strongly repressed the transcription of the dorsal organizer markers *gsc* [34] and *hhex* [49] at gastrula stage (NF10.5) and the blood island marker *hba4* (also known as *alpha-T4 globin*) [50] at stage NF28 (**Fig. 1** ***C*** and **Fig. S3** ***E***). Remarkably, similar effects were observed in embryos injected with *mNanog* (**Fig. 1** ***C***), while *OlNanog* injection did not lead to repression of *gsc* at gastrula stage (**Fig. S3** ***E***), or *hba4* at NF28 (**data not shown**). The results support the hypothesis that mammalian *Nanog* and *Xenopus ventx1/2* share functional properties, and are coherent with the observation that *OlNanog* seems to share only limited functional similarities with its mammalians orthologs [19].

### *ventx1/2* and *mNanog* Overexpression Down-regulate Specification Markers for all Germ Layers and Embryonic Territories

**Figure 2:**
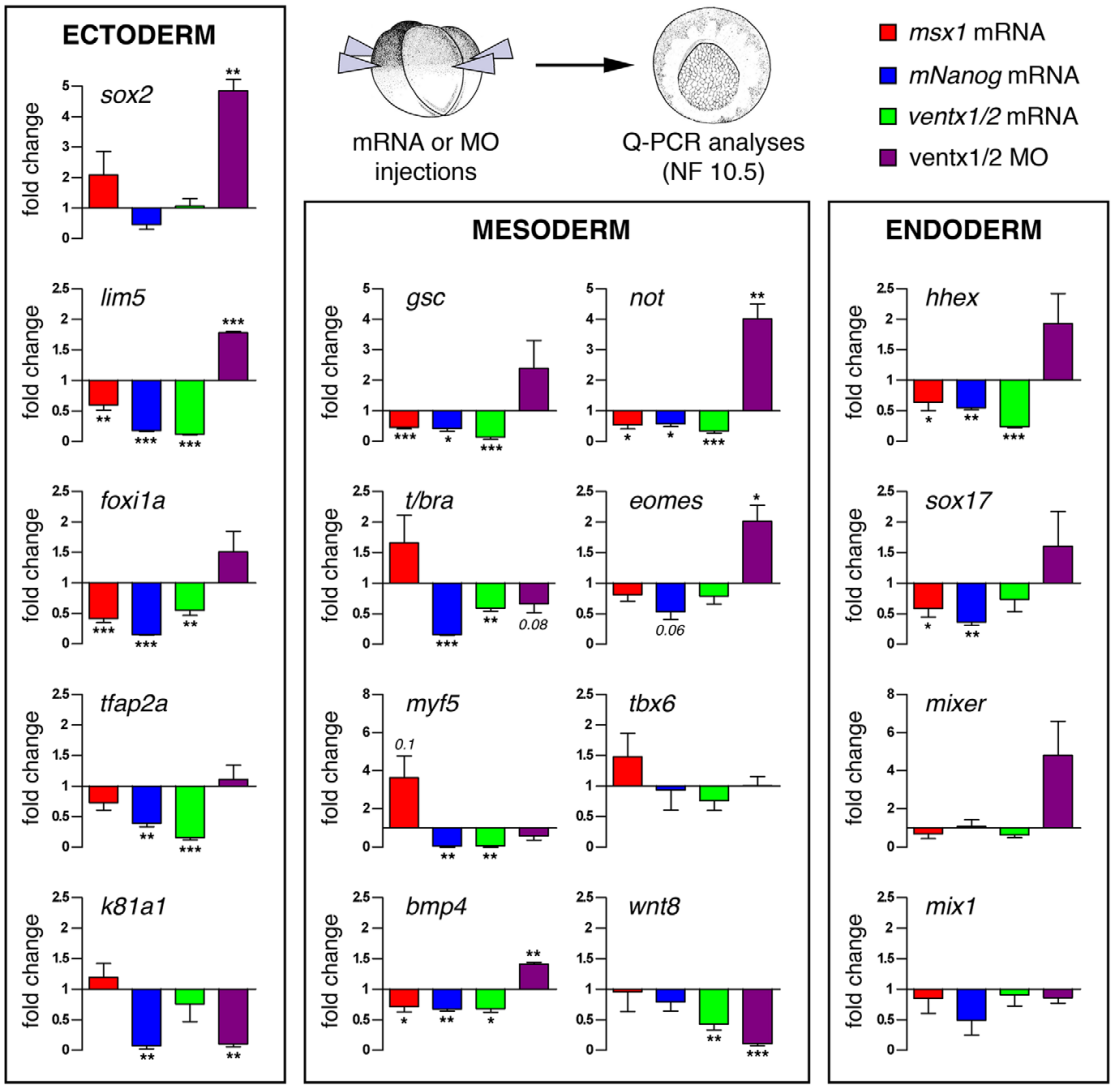
*mNanog*, *ventx1/2,* and *msx1* cause distinct effects on early patterning gene expression. For gain-of-function experiments, NF3-embryos were injected radially in all blastomeres with water, *msx1* mRNA (0.3 ng/blastomere, red), *mNanog* mRNA (0.15 ng/blastomere, blue) or *ventx1/2* mRNAs (0.5 ng/blastomere, green); For loss-of-function experiments, NF-2 embryos were injected twice radially in both blastomeres with control MO (30 ng/blastomere), or a 1∶1 mix of *ventx1/2* MOs (30 ng/blastomere, purple). All embryos were collected at stage NF10.5 and processed for RT-QPCRs. Ectodermal, mesodermal and endodermal markers were assayed (each quantification was performed at least 3 times independently). For all RT-QPCR, graphs represent means of the fold-change calculated versus the appropriate control (fldx injected embryos in cases of overexpression and control MO for *ventx1/2* knock-down) +/− s.e.m, and significance was assessed using paired t-test (*p≤0.05, **p≤0.005, ***p≤0.0005).

We next assessed whether the ventralizing effects of *ventx1/2* and *mNanog* overexpression result from similar impacts on developmental gene expression. *msx1* codes for another ventralizing NKL transcription factor and was used as a control at a dose (600 pg/embryo) known to efficiently ventralize embryos [51], [52]. Radial injections of *ventx1/2, mNanog* or *msx1* mRNAs in NF3-embryos were performed and expression levels of ectodermal, mesodermal, and endodermal markers were analyzed by RT Q-PCR at stage NF10.5 (**Fig. 2**). Strikingly, *ventx1/2* and *mNanog* repressed most markers analysed, which was not the case for *msx1*. More specifically, all three factors had comparable anti-dorsalizing activities, as revealed by the robust repression of the organizer markers *gsc, hhex*, and *not*. Remarkably, they also repressed in a similar fashion the ventral mesoderm marker *bmp4* and the early ectoderm markers *lim5* and *foxi1a*. In contrast, other genes involved in epidermis (*tfap2*), axial (*t/bra*) and paraxial mesoderm (*myf5*) commitment were significantly down-regulated by *ventx1/2* and *mNanog* but not *msx1*. Overall, *msx1*, *ventx1/2* and *mNanog* overexpression repress early markers of various cell fates in all germ layers, but marked differences appear.

We also assessed the impact of *ventx1/2* loss of function on the same markers. For this, morpholino oligonucleotides directed against *ventx1* and *ventx2* pseudoalleles (*ventx1/2* MOs) [37] or control MO were injected radially in NF3-embryos and gene expression was analyzed at stage NF10.5. We observed that *ventx1/2* knock-down led to significant overexpression of a number of genes repressed by *ventx1/2* (*not*, *lim5*, *bmp4*). This inverse regulation did not quite reach significance for all markers, perhaps reflecting the redundant activity of *ventx3s*. However, we noted that the mean level of *gsc* RNA induction in our experiment is within the range reported elsewhere [37]. Similarly, mean levels of *eomes*, *hhex*, *foxi1a*, *sox17* and *mixer* were raised, though significance was not reached. Unexpectedly, epidermal keratin (*k81a1*) was repressed both upon *ventx1/2* knock-down, and *ventx1/2* or *mNanog* overexpression. This might be explained by the fact that *ventx1/2*-deficient ectodermal cells tend to become neural, as suggested by *sox2* up-regulation. A similar line of reasoning can be applied to the ventral marker *wnt8*.

Altogether, these data suggest that *msx1*, *ventx1/2* and *mNanog* may regulate differentially early developmental networks, though causing similar ventralized phenotypes.

### *ventx1/2* and *mNanog* Repress Fate Commitment

**Figure 3:**
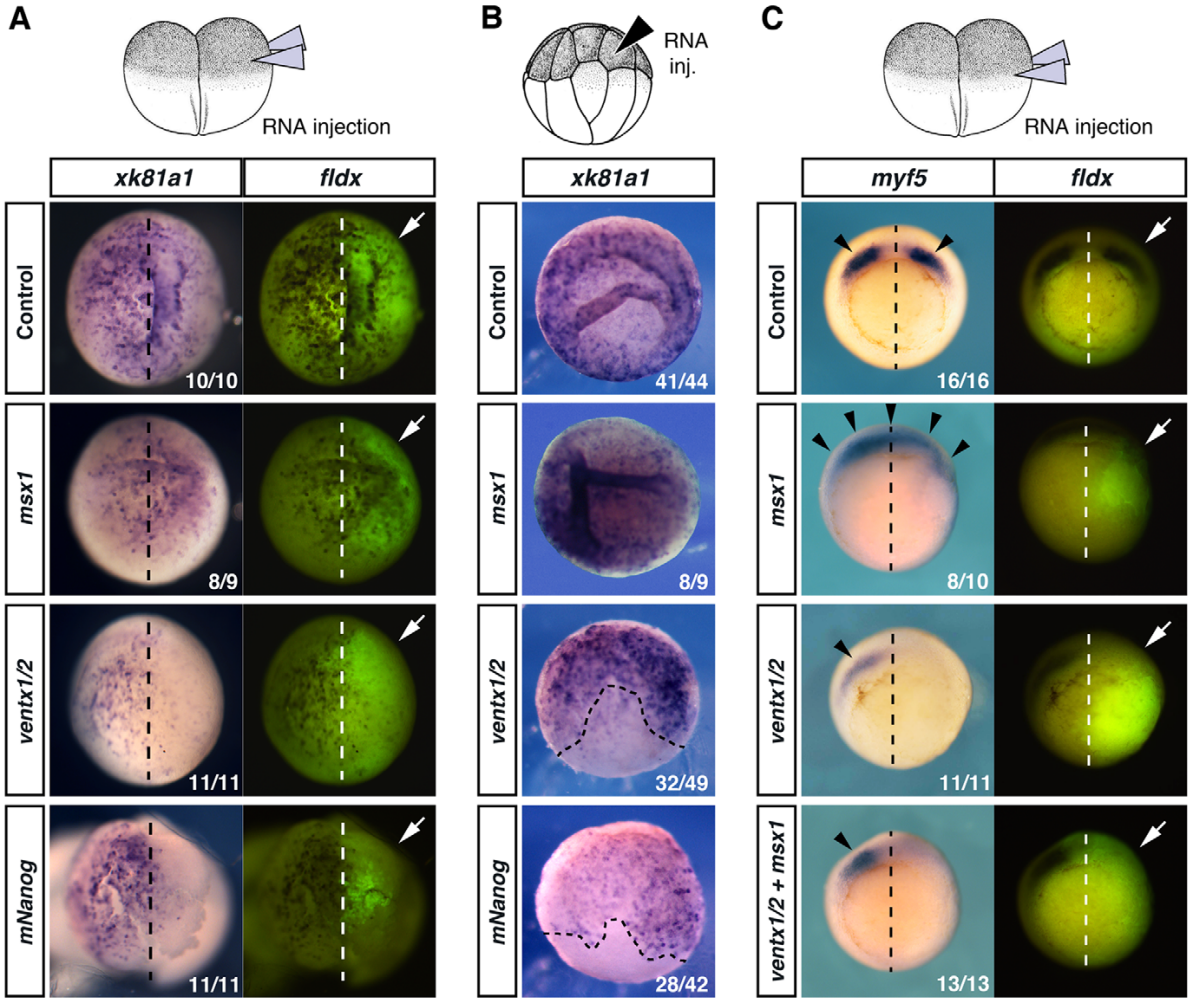
*ventx1/2* overexpression prevents multiple lineage commitment. (***A***) NF2-embryos were injected twice in one blastomere, either with *msx1* mRNAs (0.6 ng/blastomere), *ventx1/2* mRNAs (1 ng/blastomere), *mNanog* mRNA (0,3 ng/blastomere), or with water for control; fldx was used as a lineage tracer. WISH with a *k81a1* probe were performed at stage NF10.5 (left panels, animal views, dorsal side to the top). The progeny of the injected blastomere was revealed by fluorescence; white arrows indicate the injected side (right panels). (***B***) Sixteen-cell stage embryos (NF5) were injected in one AB4 blastomere with *msx1* mRNA (0.15 ng), *ventx1/2* mRNAs (0.5 ng), *mNanog* mRNA (0.15 ng), or water, collected at stage NF10.5 and processed for WISH with a *k81a1* probe (animal views). Black stripped lines mark the border between injected and uninjected domains. (***C***) NF2-embryos were injected twice in one blastomere with *msx1* mRNA (5 ng/blastomere), *ventx1/2* mRNAs (5 ng/blastomere), *ventx1/2*+*msx1* mRNAs (5 ng +5 ng/blastomere), or with water. WISH with a *myf5* probe were performed at stage NF10.5; black arrowheads point to *myf5*-expressing territories (left panels, ventral views, dorsal side to the top). The progeny of the injected blastomere was revealed by fldx fluorescence; white arrows point to the injected side (right panels).

To evaluate the above hypothesis, we focused on epidermal differentiation, which is known to require Msx1 function [52]. We injected *msx1*, *ventx1/2* or *mNanog* mRNAs either unilaterally, in one blastomere of 2-cell stage embryos (NF2, **Fig. 3** ***A***) or at the 16-cell stage (NF5, **Fig. 3** ***B***) in one AB4 blastomere fated to give rise only to epidermis. Both *mNanog* and *ventx1/2* repressed expression of the committed ectoderm marker *k81a1* in comparable fashion at NF10.5, whereas *msx1* had no effect on epidermal differentiation. This result indicates that *ventx1/2*, unlike *msx1*, do not favour, but rather impede epidermal differentiation.

To further assess if *ventx1/2* restrain commitment in *Xenopus*, the “ventralizing” activity of *msx1* was tested in the presence or excess of *ventx1/2*. Thus, *msx1* and *ventx1/2* mRNAs were injected separately or co-injected radially at the same concentration in one blastomere in NF2-embryos. Expression of the mesodermal marker *myf5* was then assessed by whole-mount *in situ* hybridization (WISH) at stage NF10.5 (**Fig. 3C**), as it is known that *msx1* positively regulates paraxial mesoderm differentiation [53]. In control embryos, *myf5* is expressed in two dorso-lateral patches around the organizer. As expected, *myf5* expression expanded into the organizer in the presence of *msx1*. Conversely, *myf5* was repressed in the *ventx1/2*-injected side, in agreement with its previously described activity on paraxial mesoderm [54]. Strikingly, joint overexpression of *ventx1/2* and *msx1* also resulted in *myf5* repression, demonstrating that *ventx1/2* are able to antagonize *msx1* activity during mesoderm commitment. Similar results were obtained using half as much *ventx1/2* mRNAs (**data not shown**), suggesting that the difference of activity between *ventx1/2* and *msx1* is qualitative rather than quantitative.

The above data suggest that commitment into specific lineages, even of ventral origin, is not possible in the presence of high levels of ventx activity.

### *ventx1/2* Knockdown Represses Pluripotency Genes and Induces Premature Commitment

As pro-differentiation genes were up-regulated in *ventx1/2* morphants (**Fig. 2**), we hypothesized that these factors are involved in the temporal restriction of commitment in early embryos. In line with this hypothesis, we detected by RT-QPCR *ventx1.2* and *ventx2.1-b* messengers in ovaries, unfertilized eggs and embryos from stages NF1 to NF10.5 (**data not shown**) and *ventx2.1-a* is known to be maternally expressed [55]. Furthermore, we found that *ventx2.1-b* is present in both animal and vegetal halves of 8-cell embryos (NF4, **Fig. S4**).

**Figure 4:**
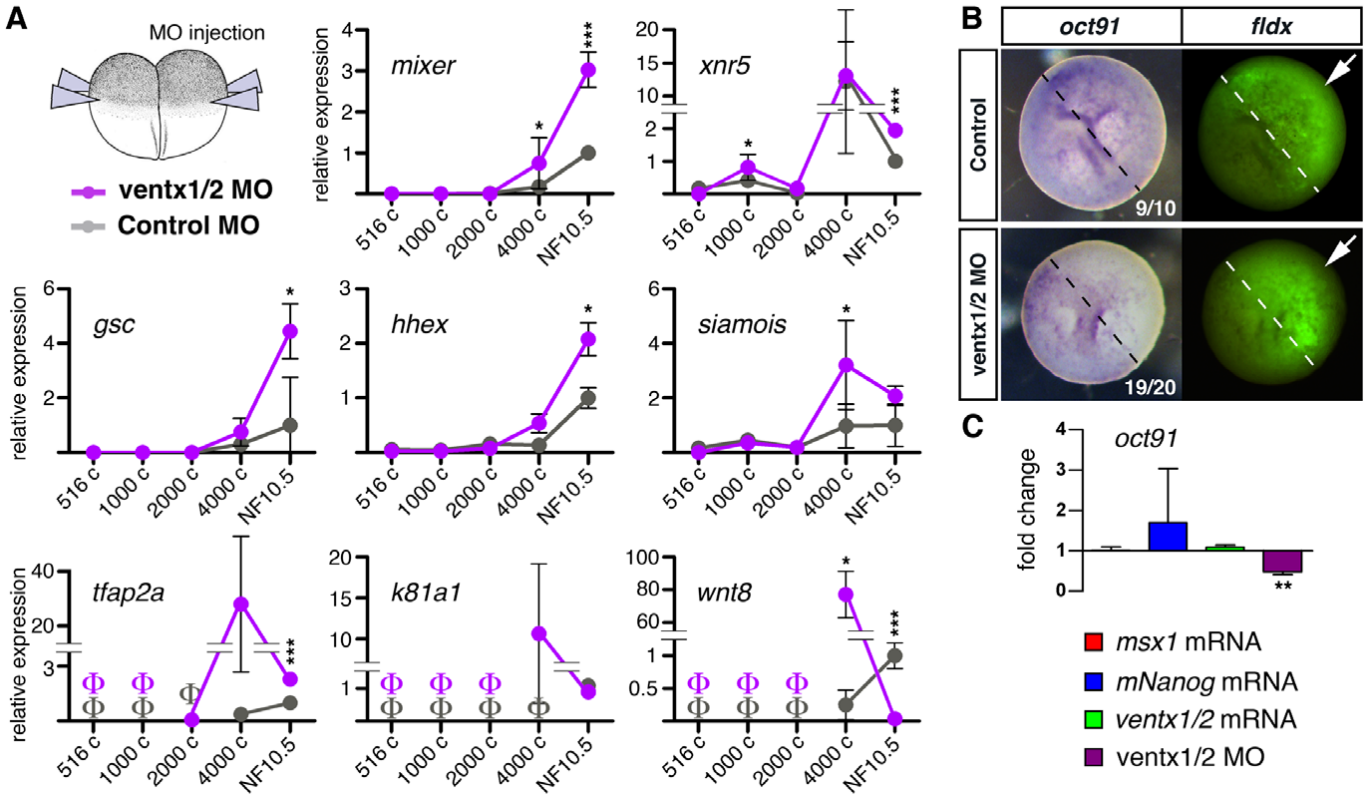
ventx1/2 activity is necessary to maintain an uncommitted cell population in early gastrulae. (***A***) NF2-embryos were injected radially twice in both blastomeres with control MO (30 ng/blastomere), or a 1∶1 mix of *ventx1/2* MOs (30 ng/blastomere). Variations of gene expression at 516-, 1000-, 2000-, 4000-cell and NF10.5 stages were assessed by RT-QPCR as in **Fig. 2.** Dorsal (*siamois*, *gsc*, *hhex*), and ventral (*wnt8*) mesendoderm, endoderm (*xnr5*, *mixer*) and ectoderm (*tfap2a*, *k81a1*) markers were monitored. Kinetic graphs represent means of fold-change relative to NF10.5 controls +/− s.e.m, and significance was assessed using paired t-test (*p≤0.05, **p≤0.005, ***p≤0.0005), and undetectable levels of transcript noted as Φ. (***B***) Animal injections were performed twice in a single blastomere NF2-embryos, using MO conditions described in (***A***); fldx was used as a lineage tracer. WISH with an *oct91* probe (left panel) were performed at stage NF10.5 and the progeny of the injected blastomere was revealed by fluorescence (right panel). Embryos are positioned with the animal side upwards; white arrows indicate the injected side. (***C***) Injections were performed using mRNA and MO conditions described in **Fig. 2**. All embryos were collected at stage NF10.5 and processed for RT-QPCRs using the pluripotency marker *oct91*. Data and graphs are presented as in **Fig. 2**.

To test whether *ventx1/2* are functionally required to restrict cellular commitment during early *Xenopus* development, we next performed RT-QPCR to monitor kinetics of expression following *ventx1/2* knockdown (**Fig. 4** ***A***). Time-course experiments revealed that in *ventx1/2* morphant embryos, expression levels of early dorsal mesendoderm (*siamois, gsc*, *hhex*), ventral mesendoderm (*wnt8*), pan-endodermal (*mixer*) and ventral ectoderm (*tfap2a*, *k81a1*) markers are higher at the 4000-cell stage when activation of zygotic transcription or “mid-blastula transition” (MBT) occurs. Remarkably, stronger expression was also observed at pre-MBT for *xnr5*, a nodal-related factor that participates in primary germ layer induction at these stages and pre-patterns the dorsal side of the embryo [56]. Overall, the expression profiles seem to be shifted to earlier time-points and to reach higher levels in *ventx1/2* morphant embryos. These results suggest that in morphant embryos, cells are no longer protected against premature commitment. Interestingly, embryos in which the POU5F1 family member *oct91* is knocked-down also fail to maintain a multipotent uncommitted cell population [25], [29]. We thus tested whether up-regulation of pro-differentiation markers in *ventx1/2* LOF is accompanied by down-regulation of this marker of the uncommitted state. The expression of *oct91* was significantly down-regulated after *ventx1/2* knockdown, as assessed by RT-Q-PCR and WISH (**Fig. 4** **,** ***B*** and ***C***). However, no significant up-regulation of *oct91* was seen following *ventx1/2* overexpression.

We conclude that *ventx1/2* activity is necessary, but not sufficient, to maintain the uncommitted status of embryonic cells during *Xenopus* early development.

### Ectopic Expression of Mouse *Nanog* but not *msx1* Rescues *ventx1/2* Morphant Embryos

**Figure 5:**
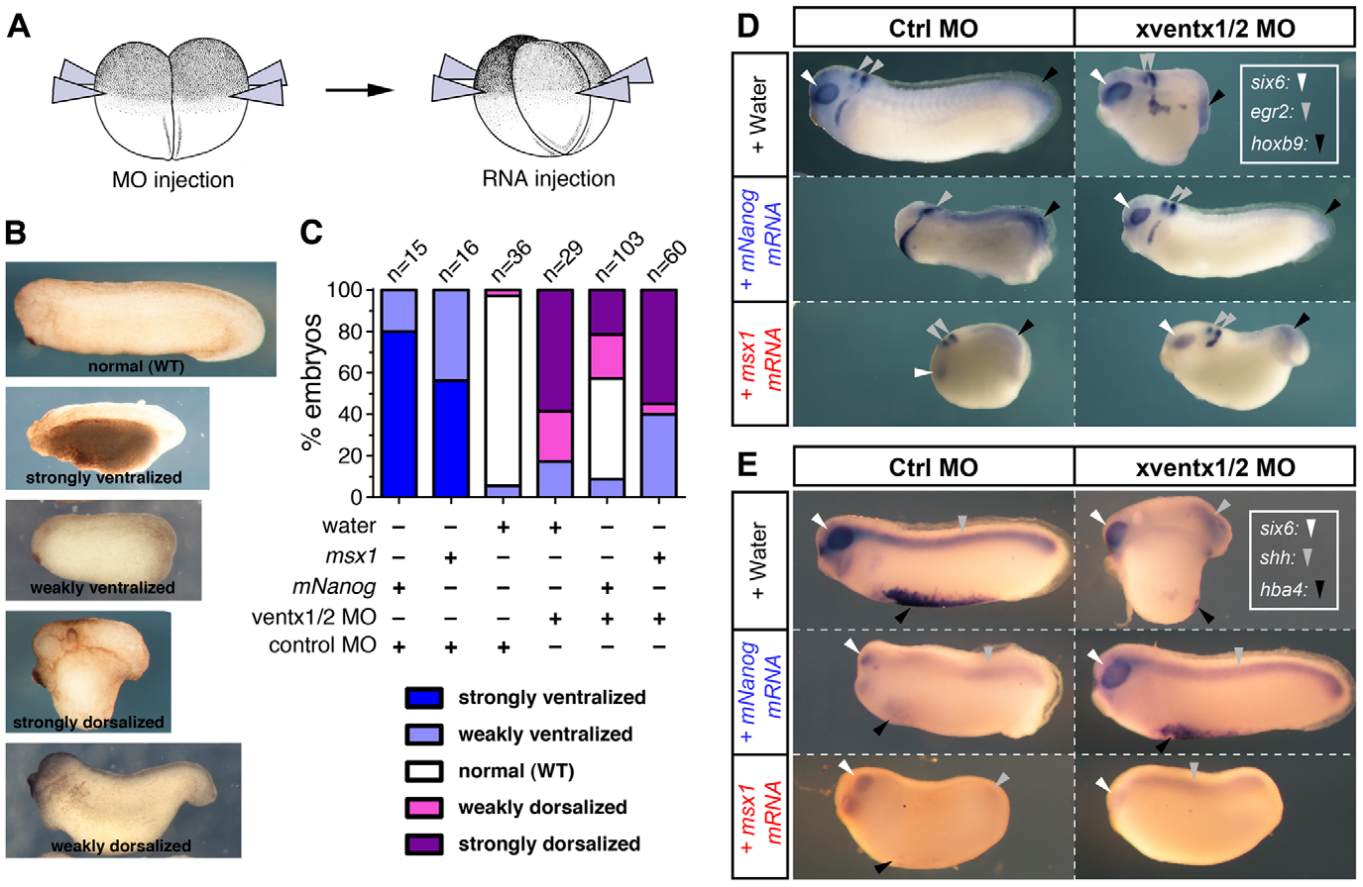
*mNanog* expression rescues specifically *ventx1/2* morphant embryos. (***A***) Two-cell stage embryos (NF2) were first injected radially twice with control MO (30 ng/blastomere), or a 1∶1 mix of *ventx1* and *ventx2* MOs (*ventx1/2* MOs; 30 ng/blastomere), and subsequently injected radially at NF3 in all blastomeres with *mNanog* mRNA (0.15 ng/blastomere), *msx1* mRNA (0.15 ng/blastomere), or with water. (***B***) Range of phenotypes observed in rescue of *ventx1/2* knockdown experiment. (***C***) Percentages observed for each phenotypic category in three independent replicates of the rescue experiment. The combined injections performed are indicated at the bottom of the graph, and the number of injected embryos for each condition is indicated on the top of each bar. NF28 embryos were processed for WISH with *six6*, *egr2* and *hoxb9* (***D***), or *six6*, *shh* and *hba4* (***E***) probes (anterior to the left, dorsal to the top).

Based on the above data, we tested the ability of *mNanog* to rescue development of *ventx1/2* knock-downed embryos. MOs directed against *ventx1/2* [37] or control MO were first injected radially in each blastomere at the 2-cell stage (NF2), followed by radial injections in all blastomeres at stage NF3 of either *mNanog* or *msx1* mRNAs (**Fig. 5** ***A***). Injections of control MO+*mNanog* and control MO+*msx1* led to a high proportion of ventralized embryos (**Fig. 5** **,** ***B*** and ***C***, about 80% and 60% respectively). As described [37], *ventx1/2* MOs injection caused dorsalization defects in 80% of embryos, whereas control injections (control MO+water) yielded 90% of normal embryos. Quite remarkably, the addition of *mNanog* mRNA to *ventx1/2* MOs produced 50% of embryos with normal morphology. Antero-posterior (**Fig. 5** ***D***) and dorso-ventral axes (**Fig. 5** ***E***) were correctly restored in such embryos, as revealed by whole mount *in situ* hybridization for markers of notochord (*shh*), ventral blood islands (*hba4*), spinal cord (*hoxb9*) and brain (*six6* and *egr2,* also known as *optx2* and *krox-20* respectively). In contrast, *msx1* overexpression failed to restore a normal morphology in *ventx1/2* morphant embryos. In this condition, about 60% of embryos remained dorsalized and about 40% became ventralized, confirming that *msx1* ventralizes embryos through mechanisms distinct from the ones induced by *ventx1/2* and *mNanog* (**Fig. 5** ***E***). Altogether, these results demonstrate that *mNanog* is able to substitute for *ventx1/2* in *Xenopus* development and that this effect is specific.

## Discussion

In this study, we report a number of properties of *ventx1/2* in the *Xenopus* embryo that call for a reassessment of their biological role. Although *ventx1/2* and *msx1* are thought to be ventralizing regulators, their overexpression has distinct effects. *msx1* suppresses dorsal but not ventral markers [52,53 and our results], whereas *ventx1/2* suppress both dorsal and ventral markers in mesoderm and in epidermis [50,55, and our results]. Consequently, ventx1/2 cannot be considered *bona fide* ventralizing factors. Instead, we propose that ventx1/2 are guardians of developmental potential in the early embryo, a function that is necessary to achieve proportioned and progressive building of the body.

Supporting this view, we found that, in gastrulae, increased ventx1/2 activity represses the expression of transcription factors involved in early cell commitment in all germ layers, including a significant down-regulation of *hhex*, *gsc*, *not*, *bmp4*, *t/bra, wnt8, lim5*, *foxi1a*, *tfap2a,* and *myf5* (**Fig. 1** and **Fig. 2**). Conversely, knockdown of *ventx1/2* results in the significant up regulation of the differentiation markers *sox2*, *lim5*, *bmp4*, *not*, *eomes* (**Fig. 2**). Further, we show that ventx1/2 are necessary to maintain normal expression levels of the well-identified pluripotency effector *oct91* during gastrulation (**Fig. 4** **,** ***B*** and ***C***). Thus, *ventx1/2* appear to be involved in an active mechanism protecting blastula/gastrula cells from the pro-differentiation cues that establish germ layers and pattern the embryonic axis. The earliest role of *ventx1/2* would therefore not be the establishment of ventral identity, but rather to prevent premature commitment, similar to *Xenopus pou5f1s* [25], [29], [44], [57], [58]. In line with this hypothesis, it is important to note i) that cells in early *ventx1/2* expressing territories (ectoderm and ventral mesoderm) remain multipotent until late gastrulation [59], [60], ii) that active clearance of ventx proteins coincides with loss of multipotency at mid-gastrula stages [61], iii) that post-gastrula *ventx2.1* expression territories coincide with stem cell-containing niches such as the dorsal ciliary margin of the eye [47] and the tailbud [62] and iv) that *ventx2.1* is re-expressed together with *oct91* in *Xenopus* somatic cells reprogrammed to an iPS-like state *in vivo* [63].

Our work thus highlights the role of *ventx1/2* in linking cell commitment to embryonic axis patterning and agrees with experimental and theoretical works suggesting that the dorso-ventral genetic system, to which *ventx1/2* belong, functions primarily as a regulator of the timing of cell commitment [64]. Indeed, according to our hypothesis, ventx1/2 would maintain early embryonic cells in an undetermined state and limit their competence to respond to differentiation-inducing signals, as NANOG does to maintain pluripotency in mammalian embryos [16], [65]. The ability of *mNanog* to rescue the *ventx1/2* morphant phenotype in *Xenopus* embryos strongly supports this contention (**Fig.** **5**). In our re-interpretation of their role, ventx1/2 factors, as regulators of timing of commitment, control the progressive allocation of embryonic cells to the developing body axis. Loss of *ventx1/2* allows cellular commitment and most cells precociously adopt dorsal and anterior positional identities, similar to the cells that first become negative for *ventx1/2* in normal embryos. Consequently, the pool of cells available to build posterior territories is depleted, resulting in minute trunk-tail structures [37]. Conversely, ectopic ventx1/2 activity represses early commitment factors and thus causes dorso-anterior truncations [34]. As such embryos do develop posterior structures, we surmise that the uncommitted cellular state is only transient, in agreement with the reported loss of cellular competence at the end of gastrulation in normal embryos. Importantly, our data supports a role for *ventx1/2* in restricting cell commitment starting at pre-MBT stages. Recent work underlines the importance of Wnt/βcat and Nodal signalling in priming cells for induction of mesendoderm and establishment of dorsal identity as early as the 1000-cell stage, well before MBT [56], [66]. Here, we show that knockdown of *ventx1/2* results in premature and/or increased expression of a number of developmental genes including the *nodal*-related *xnr5*, as well as the dorsal organizer genes *hhex*, *gsc* and *siamois* at or before the MBT (**Fig.** **4** ***A***). Taken together, the evidence presented here supports the concept that control of developmental potential is a strategy that ensures correct germ layer formation and body patterning, common to all gnathostomes [25].

Our proposed role of *ventx1/2* in the control of cellular differentiation echoes earlier studies performed by W. Knöchel and collaborators [44]. Interestingly, these researchers recently tested the hypothesis that *Xenopus ventxs* could be functional homologs of *Nanog* [20]. However, their results contrast sharply with ours, as they did not observe rescue of morphant *ventx1/2* embryos by *mNanog*. The reason for this discrepancy is unclear but may possibly reflect differences in experimental setup. The positive evidence of rescue of *ventx1/2* knockdown by *mNanog* in this paper supports the view that *ventx* and *Nanog* genes are related and that *Xenopus ventx1/2* and mammalian *Nanog* serve comparable developmental functions through the regulation of overlapping transcription programs (see **Table S2**). Indeed, while genetic responses induced by *mNanog* and *ventx1/2* overexpression do not perfectly match at early stages (NF10.5, **Fig. 2**), these differences seem to be buffered by the regulation networks at work during germ layer specification and embryonic patterning, up to the point that *mNanog* is able to substitute for *ventx1/2* to generate morphologically normal larvae (**Fig. 5**).

Other amphibians possess *Nanog* orthologs [21], [22], raising the question of whether the role of *ventxs* in developmental potential maintenance is ancestral, or is an innovation specific to *Xenopus*. Functional data strongly support the ancestrality of *ventxs* involvement in this process, since teleost and *Xenopus ventxs* serve the same function during development [45], [67], [68]. Unfortunately, data concerning amniote *Ventx* genes is scarce, probably because they are absent from the genome of the mouse, the main experimental model in this taxon. Partial functional redundancy between *Ventx* and *Nanog* might explain the loss of the former in rodents and of the latter in *Xenopus*. Some indirect evidence supports this notion in mammals. The human *ventx* ortholog (*VENTX*) located next to the stem-cell marker *UTF-1*, shares features with its counterparts in *Xenopus* and fish [69]. Both human and *Xenopus ventx* orthologs [70] are direct targets of POU5F1 transcription factors [44], [71], [72], and human *VENTX* displays ventralizing activity in zebrafish embryos [69]. As mentioned earlier, *VENTX* retropseudogenes are unusually frequent in the human genome [40], a feature that is proposed to be a specific signature of genes involved in pluripotency maintenance such as *POU5F1* and *NANOG* [41]. In line with this idea, *VENTX* is co-expressed with *NANOG* and *POU5F1* in pluripotent-embryonal carcinomas [73], a subtype of human male germ cell tumours constituted of cells highly similar to early zygotic and ES cells [74], and these three genes are strongly down-regulated when tumour cell differentiation is forced *in vitro* [73]. Furthermore, a genome-wide RNA interference screen has shown that in human ES cells, *VENTX* or *NANOG* knockdown results in reduced expression of a POU5F1-GFP reporter construct in a comparable way (see Supplemental Information in [75]). Finally, *VENTX* expression is under the control of the POU5F1/SOX2/NANOG triumvirate, (http://biit.cs.ut.ee/escd/index.cgi?gene=ventx [72], [76]) suggesting that it may be part of the human pluripotency-regulating network [72], [76].

In conclusion, our data strongly support the concept that *ventx1/2* act as guardians of high developmental potential during *Xenopus* early development. We propose that this role of *Ventx* genes is ancestral and conserved in gnathostomes, a question for future research with high biomedical relevance.

## Materials and Methods

### Ethics Statement

The care and treatment of animals used in this study were in accordance with institutional and national guidelines (Commission de Génie Génétique, “Direction Départementale des Services Vétérinaires”, European Union Directive 2010/63, registered as No. 4654 for the agreement decision, and as No. B 75-05-01 for the vertebrate living animals experimentation; this commission specifically approved this study).

### In Silico Screening

Homeodomain sequences from all reported *Nanog* genes were retrieved from public repositories (http://www.ncbi.nlm.gov/; http://www.ensembl.org/) and used as queries to perform several rounds of TBLASTN screening on the *Xenopus tropicalis* genome assembly (http://genome.jgi-psf.org/Xentr4/Xentr4.home.html), as well as on available expressed sequence tags and cDNA sequences from *Xenopus tropicalis* and *Xenopus laevis* (http://www.ncbi.nlm.gov/). Control searches using the same queries to screen published gnathostome genomes allowed the identification of at least one likely candidate in each case.

### Reverse Transcriptase - PCR Screening

Total RNAs were extracted from *Xenopus laevis* ovaries, unfertilized and fertilized eggs (NF1), blastulae (NF8) and early gastrulae (NF10.5). The RT-PCR protocol and primers used are detailed in supporting information.

### Phylogenetic and Conservation Analyses

The methodologies used to perform molecular phylogenetic and conservation analyses of the NKL family are detailed in supporting information.

### *Xenopus* Embryo Manipulations

Sacrifices and animal studies were conducted according to the principles and procedures described in Guidelines for Care and Use of Experimental Animals. *Xenopus laevis* were obtained by *in vitro* fertilization and staged according to Nieuwkoop and Faber [48] and cultured according to Slack *et al.* [77]. Embryos were injected with RNAs and/or morpholino oligonucleotides (MOs) as described in the relevant figure legends. We determined that the lethal dose for *mNanog* is 1 ng per embryo (**data not shown**). Dextran fluorescein (fldx; Molecular Probes) was used as a lineage label. In order to rule out possible interference of MOs mixed with mRNAs before injection, we performed rescue assays through injections of MOs at the 2-cell stage (NF2), followed by mRNAs injections at the 4-cell stage (NF3). All injections were performed at least three times to assess reproducibility.

### *In vitro* Translation and Morpholino Oligonucleotides

Synthetic capped mRNAs were transcribed with the mMessage mMachine SP6 kit (Ambion) using the following templates: pCS2+-Vent1 and pCS2+-Xbr1b, both linearised with NotI (gifts of N. Papalopulu, University of Manchester, UK and collectively referred to as *ventx1/2* in this work); pSP64T-xMsx1, linearised with EcoRI; pCS2+OlNanog, linearised with SacII (Gift of JL. Mullor, Centro de Investigación Príncipe Felipe, Valencia, Spain). To express *mNanog*, the ORF of a commercial clone (Geneservice) was PCR amplified and cloned into pCS2+, linearised with NotI and transcribed with mMESSAGE mMACHINE SP6 Kit (Ambion). Previously described morpholino oligonucleotides (MOs) directed against *ventx1* and *ventx2* pseudoalleles [37] were obtained from GeneTools.

### Whole Mount *in situ* Hybridization

Injected embryos were processed for whole-mount in situ hybridization (WISH) with digoxigenin-labelled probes (Roche) using standard procedures and staining was done with BM purple (Roche). Embryos were bleached with hydrogen peroxide 4% (Carlo Erba Reagenti) and photographed with a MZ16F binocular (Leica).

### Real-time Quantitative PCR and Statistical Analyses

For real-time quantitative PCR (RT-QPCR) total RNAs were extracted from 10 post-MBT (**Figs. 2** and **4** ***C***) or 5 pre-MBT (**Fig. 4** ***A***) embryos using RNeasy Plus Mini kit (Qiagen) and reverse transcribed using Superscript II reverse transcriptase (Invitrogen). Independent biological replicates were collected and RT-QPCR reactions were performed in duplicate for each sample using Power SYBR® master mix on a 7300 Real-Time PCR System (Applied Biosystems), following manufacturer recommendations. Primers (MWG Biotech) were described in previous publications or designed using Primer Express Software (Applied Biosystems), the relevant sequences and references are listed in **Table S3**. Primers for the housekeeping gene *DNA elongation factor type 1 α* (*ef1a1*) or *ornithine decarboxylase 1* (*odc1*) were used as loading control for samples collected post-MBT (**Figs. 2** and **4** ***C***) and pre-MBT (**Fig. 4** ***A***), respectively. Ct data were collected using 7300 system software (Applied Biosystem) and analyzed using Excel (Microsoft). For **Figs**. **2** and **4** ***C***, the Ct for each technical duplicate was averaged and normalized against *ef1a1*. Variations of expression were quantified using the ΔΔCts method, using the control condition as reference for each experimental replicate and fold changes were computed as 2^ΔΔCt^. Data from independent experiments were averaged, plotted and significance was assessed (two-way paired Student’s t-test) using Prism 5.03 (GraphPad). For **Fig.** **4** ***A*** ***,*** the Ct were normalized against *odc1*. Levels of expression were quantified using the ΔCts method averaged, plotted and significance was assessed (two-way paired Student’s t-test) using Prism 5.03 (GraphPad).

## Supporting Information

Figure S1 **Phylogenic reconstruction of the NKL group homeodomains relationships using Maximum Likelihood.** (***A***) Global view of an unrooted maximum likelihood tree obtained with the homeodomain sequences of all known NKL members found in the genomes of the fly, amphioxus and a representative selection of vertebrates (see Supporting Information for Extended Experimental Procedures). NKL families are highlighted in different shades of grey except for NANOG (red) and VENTX (blue). Relationships between NKL families remain elusive; however all are monophyletic and well supported by bootstrap analysis with three exceptions: the NK4 (paraphyletic) VENTX (polyphyletic) and NANOG (monophyletic, but poorly supported, bootstrap: 54,4%). (***B***) Close-up of the region of the tree where most VENTX orthologs are found. (***C***) Close-up of the region of the tree containing the monophyletic NANOG group. Note that amphioxus VENTX homeodomains (VENT1 *Branchiostoma floridae* and VENT2 *Branchiostoma floridae*) are found at the root of the NANOG subtree. However, this association is not supported by bootstrap analysis (bootstrap: 18,7%) and the interpretation of amphioxus VENTXs as NANOG orthologs is at odds with the literature [39]. Both NANOG and the main VENTX group have longer branches than typical NKL-class members (e.g. NK1 and LBX groups on panels ***B*** and ***C***, see also **Table S1**).
(TIF)

Figure S2 **Determination of lethal doses of** ***mNanog*** **and** ***OlNanog.*** NF3-embryos were injected radially in all blastomeres, with water, *mNanog* mRNA or *OlNanog* mRNA at multiples doses and embryonic lethality was assessed at late blastula (NF9) and early tadpole (NF31). The doses indicated correspond to the total amount of mRNA injected per embryo. (***A-C***) Representative NF9 embryos observed in the indicated conditions (top panels), an arrow points to the embryos shown at greater magnification (bottom panels). The number of embryos injected is indicated. (***D-E***) Percentage of lethality observed at NF9 and NF31 after *mNanog* and *OlNanog* injection, respectively. The numbers of dead and living embryos observed at both time points is given under the graphs. Note that a dose of 1,2 ng of *mNanog* results in 100% embryonic lethality at NF9, while 0,6 ng (half the lethal dose) had no toxic effect; hence this condition was retained for further study. Conversely, *OlNanog* overexpression led to increased lethality beyond the 2 ng injection condition of *OlNanog* RNA (dotted line). Embryo death arose from 5 ng injected embryos, and about 40% or 100% lethality was observed at NF31 for the 5 ng or the 10 ng conditions respectively. Hence the condition with 1,2 ng injected embryos was retained for further study.
(TIF)

Figure S3 ***OlNanog*** **overexpression leads to phenotypes that strongly differ from those observed upon** ***mNanog*** **or** ***ventx1/2*** **overexpression.** (***A***) NF3 embryos were injected radially with *OlNanog* mRNA (0.6 ng, 1.2 ng, 2 ng and 5 ng./embryo), or with water for control. Representative phenotypes observed at early tadpole stage (NF31) are shown (lateral views, anterior to the left, dorsal to the top). (***B***) Percentages of observed phenotypes for the different *OlNanog* mRNAs doses assayed. Across the whole range of concentration used, the phenotypes obtained in *OlNanog*-injected embryos strongly differed from those resulting from *mNanog* overexpression (***C*** and ***D***). No cues of ventralization were observed as seen with *mNanog*-injected embryos (see black arrowheads in ***C***), the embryos retaining distinguishable head structures. The main effect was a shortened axis, resulting from defects in blastopore closure (see white arrowheads in ***A***).
(TIF)

Figure S4 ***ventx2.1*** **mRNAs are present in animal and vegetal cells of 8-cell stage** ***Xenopus*** **embryos.** (***A***) 8-cell stage *Xenopus* embryos were separated in animal and vegetal halves, which were separately processed for RT-QPCR. (***A***) *ventx2.1* (green) mRNA abundance in the two territories was estimated relative to the *odc* loading control marker, while *vegt* (purple) was used as a positive control. (***B***) As expected, we observed that *vegt* mRNA is almost exclusively localised in the vegetal blastomeres, while in contrast *ventx2.1* mRNA is predominantly found in animal blastomeres but is also significantly present in vegetal blastomeres.
(TIF)

Table S1 **Nanog and Ventx homeodomains are less conserved than other NKL families.** For each NKL family conserved among vertebrates (1^st^ column) the homeodomains (HDs) of all *Homo sapiens*, *Xenopus tropicalis*, *Danio rerio* and *Takifugu rubripes* paralogs were retrieved (see Supporting Information S1 for Extended Experimental Procedures). When a given paralog was unknown in a given species but present in a closely related one, this alternate sequence was used instead. More specifically: (£) EMX1 being unknown in *Takifugu rubripes*, the *Tetraodon nigroviridis* sequence was used; (&) NANOG being unknown in *Xenopus* species the *Ambystoma mexicanum* sequence was used. For each group of orthologs, the percentage of identity along the HD of the four relevant sequences was computed. For families with multiple paralogs, only the least conserved are shown here (2^nd^ column). The consensus sequence and percentage of identity thus obtained are indicated (3^rd^ and 4^th^ columns). The VENTX and NANOG families (in bold) present the lowest sequence identity in the HD, and are the only NKL families for which numerous processed pseudogenes are found in the human genome (5^th^ column) [40,78]. This similarity extends to functional properties (see **Table S2**).
(TIF)

Table S2 **Mammalian** ***Nanog*** **and** ***Xenopus ventxs*** **share striking functional similarities.** Mammalian *Nanog* (left) and *Xenopus ventx1/2* (right) are “regulated by” (A and B), “regulate” (C) and “interact” (D) with homologous pathways, transcription factors, genes and proteins, respectively. Most of these factors are known to regulate pluripotency and/or cell commitment and differentiation in mammals (indicated by P/C), while their counterparts in frog are known to be involved in dorso/ventral patterning during embryogenesis (indicated by D/V). References 78–110 are listed as Supplemental References in Supporting Information.
(TIF)

Table S3 **Primer pairs used for RT-QPCR experiments in this study.** For each primer pair, the forward and reverse sequences are listed, as well as the original publications (references 111–125 are listed as Supplemental References in Supporting Information).
(TIF)

Supporting Information S1 **The Supporting Information file contains Extended Experimental Procedures and Supplemental References.**
(DOC)
