# Supplementary figures and images for: Ventx Factors Function as Nanog-Like Guardians of Developmental Potential in Xenopus
Source: PLoS One. 2012 May 14;7(5):e36855. doi: 10.1371/journal.pone.0036855 (PMC3351468; doi:10.1371/journal.pone.0036855)

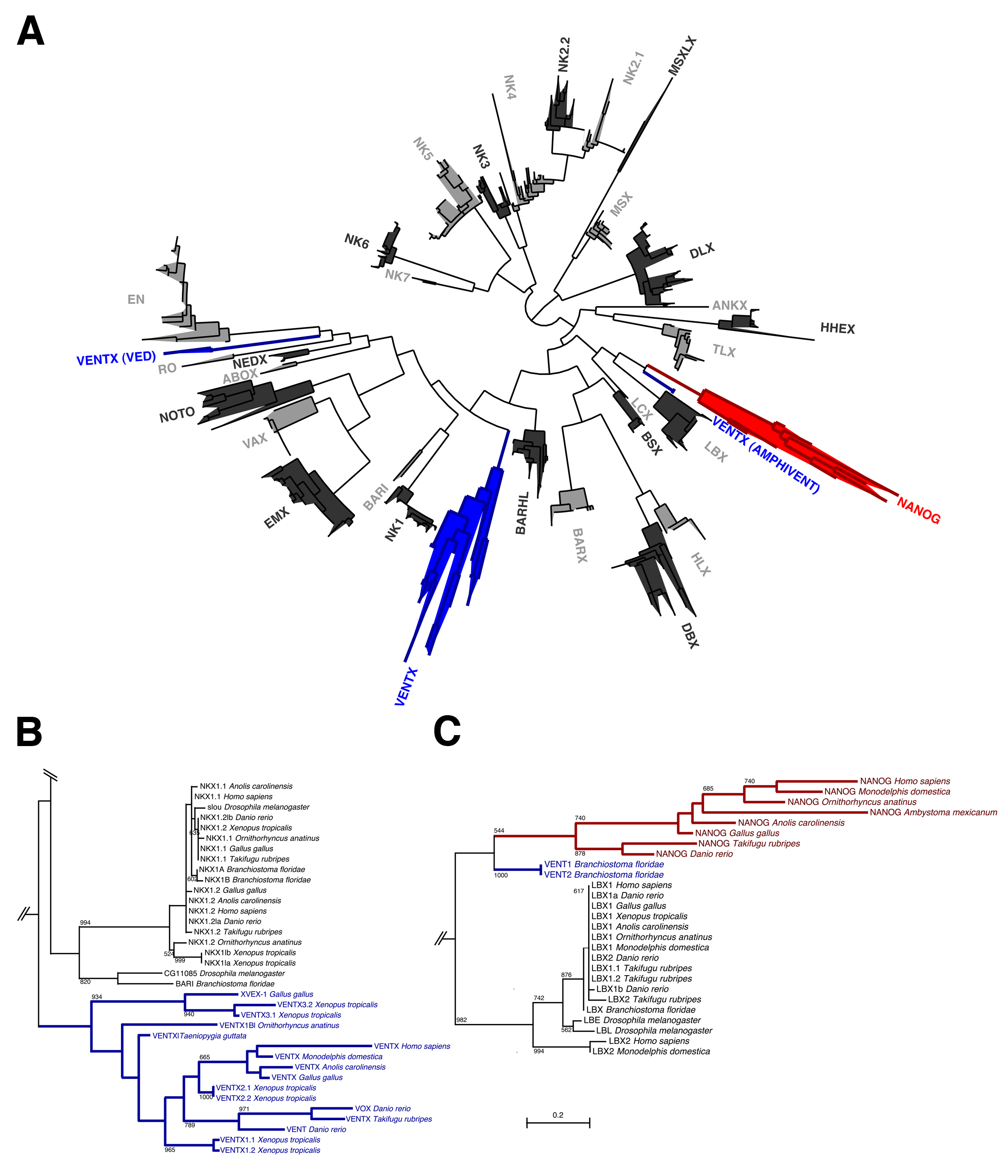

Supplement: Figure S1 — Phylogenic reconstruction of the NKL group homeodomains relationships using Maximum Likelihood. (A) Global view of an unrooted maximum likelihood tree obtained with the homeodomain sequences of all known NKL members found in the genomes of the fly, amphioxus and a representative selection of vertebrates (see Supporting Information for Extended Experimental Procedures). NKL families are highlighted in different shades of grey except for NANOG (red) and VENTX (blue). Relationships between NKL families remain elusive; however all are monophyletic and well supported by bootstrap analysis with three exceptions: the NK4 (paraphyletic) VENTX (polyphyletic) and NANOG (monophyletic, but poorly supported, bootstrap: 54,4%). (B) Close-up of the region of the tree where most VENTX orthologs are found. (C) Close-up of the region of the tree containing the monophyletic NANOG group. Note that amphioxus VENTX homeodomains (VENT1 Branchiostoma floridae and VENT2 Branchiostoma floridae) are found at the root of the NANOG subtree. However, this association is not supported by bootstrap analysis (bootstrap: 18,7%) and the interpretation of amphioxus VENTXs as NANOG orthologs is at odds with the literature [39]. Both NANOG and the main VENTX group have longer branches than typical NKL-class members (e.g. NK1 and LBX groups on panels B and C, see also Table S1). (TIF) [file pone.0036855.s001.tif]

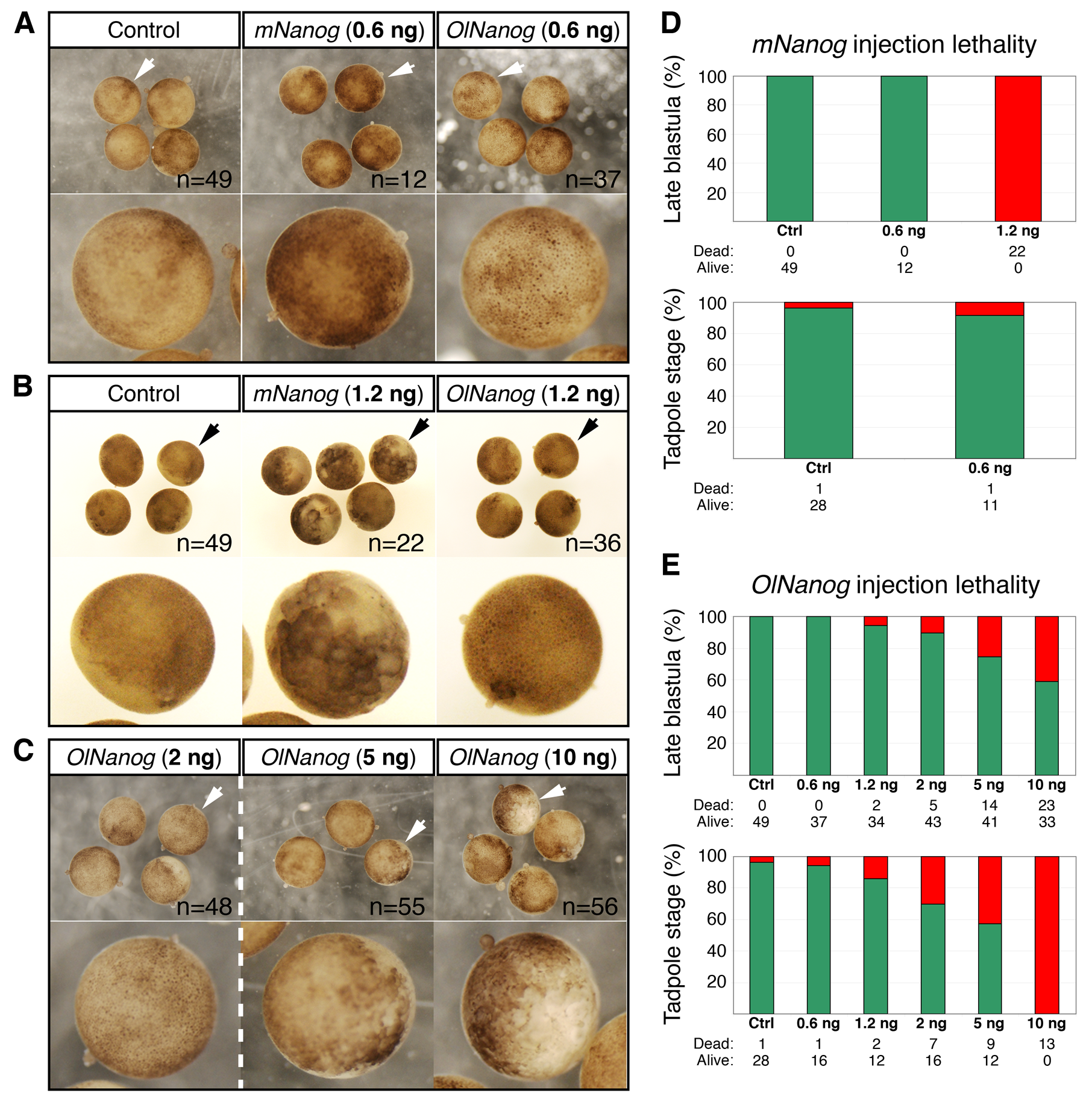

Supplement: Figure S2 — Determination of lethal doses of mNanog and OlNanog. NF3-embryos were injected radially in all blastomeres, with water, mNanog mRNA or OlNanog mRNA at multiples doses and embryonic lethality was assessed at late blastula (NF9) and early tadpole (NF31). The doses indicated correspond to the total amount of mRNA injected per embryo. (A-C) Representative NF9 embryos observed in the indicated conditions (top panels), an arrow points to the embryos shown at greater magnification (bottom panels). The number of embryos injected is indicated. (D-E) Percentage of lethality observed at NF9 and NF31 after mNanog and OlNanog injection, respectively. The numbers of dead and living embryos observed at both time points is given under the graphs. Note that a dose of 1,2 ng of mNanog results in 100% embryonic lethality at NF9, while 0,6 ng (half the lethal dose) had no toxic effect; hence this condition was retained for further study. Conversely, OlNanog overexpression led to increased lethality beyond the 2 ng injection condition of OlNanog RNA (dotted line). Embryo death arose from 5 ng injected embryos, and about 40% or 100% lethality was observed at NF31 for the 5 ng or the 10 ng conditions respectively. Hence the condition with 1,2 ng injected embryos was retained for further study. (TIF) [file pone.0036855.s002.tif]

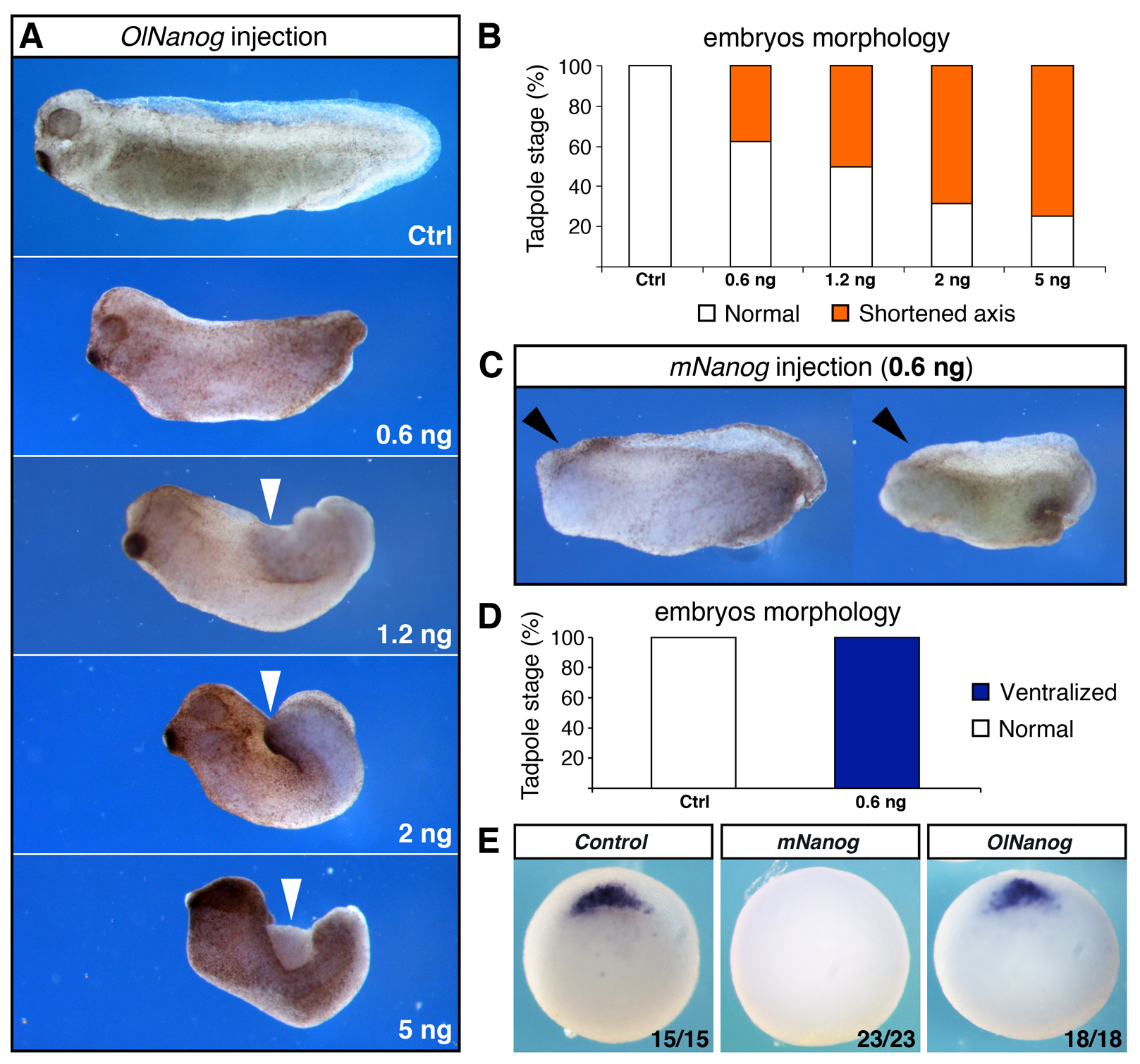

Supplement: Figure S3 — OlNanog overexpression leads to phenotypes that strongly differ from those observed upon mNanog or ventx1/2 overexpression. (A) NF3 embryos were injected radially with OlNanog mRNA (0.6 ng, 1.2 ng, 2 ng and 5 ng./embryo), or with water for control. Representative phenotypes observed at early tadpole stage (NF31) are shown (lateral views, anterior to the left, dorsal to the top). (B) Percentages of observed phenotypes for the different OlNanog mRNAs doses assayed. Across the whole range of concentration used, the phenotypes obtained in OlNanog-injected embryos strongly differed from those resulting from mNanog overexpression (C and D). No cues of ventralization were observed as seen with mNanog-injected embryos (see black arrowheads in C), the embryos retaining distinguishable head structures. The main effect was a shortened axis, resulting from defects in blastopore closure (see white arrowheads in A). (TIF) [file pone.0036855.s003.tif]

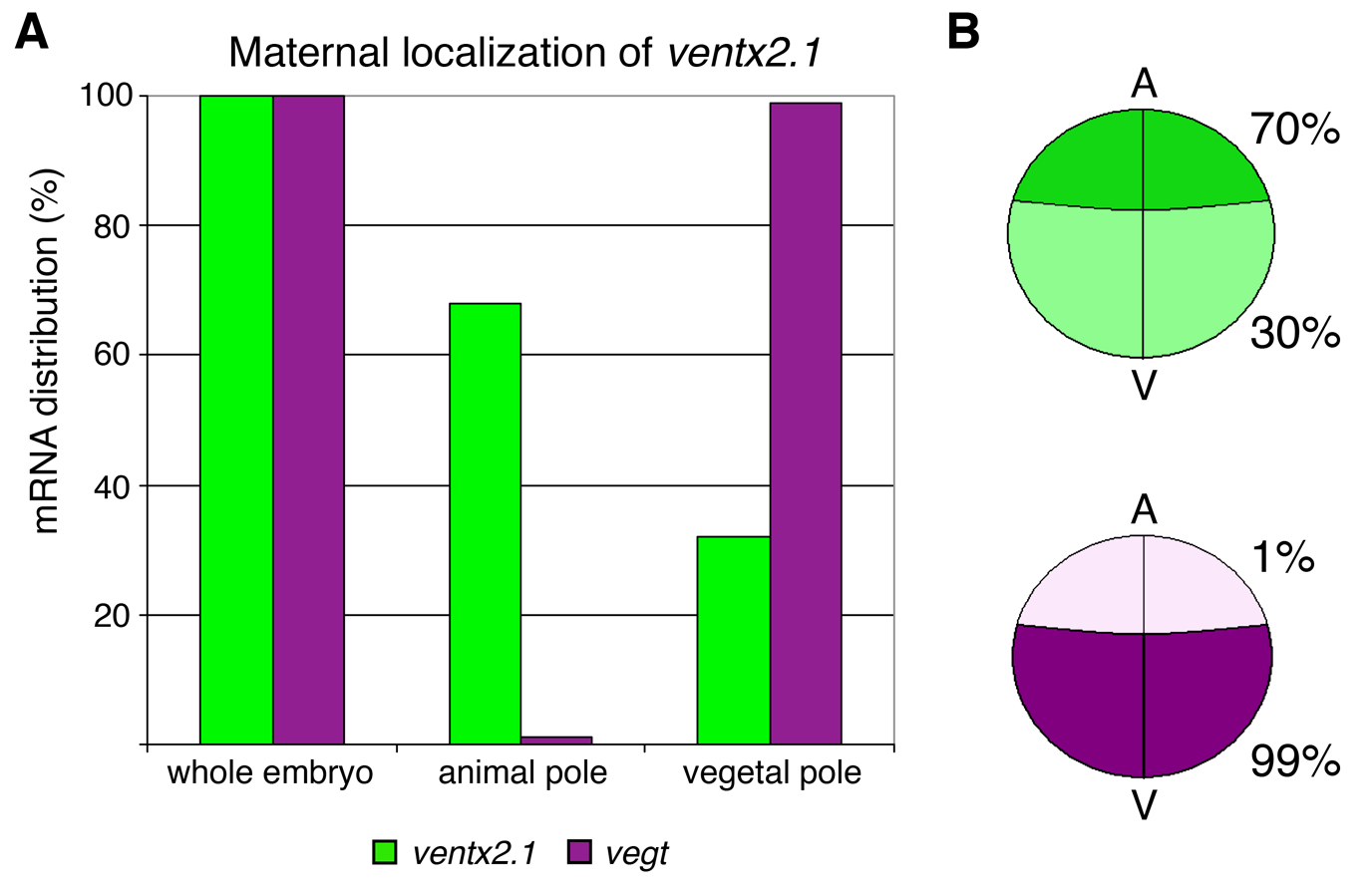

Supplement: Figure S4 — ventx2.1 mRNAs are present in animal and vegetal cells of 8-cell stage Xenopus embryos. (A) 8-cell stage Xenopus embryos were separated in animal and vegetal halves, which were separately processed for RT-QPCR. (A) ventx2.1 (green) mRNA abundance in the two territories was estimated relative to the odc loading control marker, while vegt (purple) was used as a positive control. (B) As expected, we observed that vegt mRNA is almost exclusively localised in the vegetal blastomeres, while in contrast ventx2.1 mRNA is predominantly found in animal blastomeres but is also significantly present in vegetal blastomeres. (TIF) [file pone.0036855.s004.tif]
