# Supplementary material for: Ventx Factors Function as Nanog-Like Guardians of Developmental Potential in Xenopus
Source: PLoS One. 2012 May 14;7(5):e36855. doi: 10.1371/journal.pone.0036855 (PMC3351468; doi:10.1371/journal.pone.0036855)
Supplement: Table S1 — Nanog and Ventx homeodomains are less conserved than other NKL families. For each NKL family conserved among vertebrates (1st column) the homeodomains (HDs) of all Homo sapiens, Xenopus tropicalis, Danio rerio and Takifugu rubripes paralogs were retrieved (see Supporting Information S1 for Extended Experimental Procedures). When a given paralog was unknown in a given species but present in a closely related one, this alternate sequence was used instead. More specifically: (£) EMX1 being unknown in Takifugu rubripes, the Tetraodon nigroviridis sequence was used; (&) NANOG being unknown in Xenopus species the Ambystoma mexicanum sequence was used. For each group of orthologs, the percentage of identity along the HD of the four relevant sequences was computed. For families with multiple paralogs, only the least conserved are shown here (2nd column). The consensus sequence and percentage of identity thus obtained are indicated (3rd and 4th columns). The VENTX and NANOG families (in bold) present the lowest sequence identity in the HD, and are the only NKL families for which numerous processed pseudogenes are found in the human genome (5th column) [40,78]. This similarity extends to functional properties (see Table S2). (TIF) [file pone.0036855.s005.tif]

**TABLE S1: Nanog and Ventx homeodomains are less conserved than other NKL families.**

| **NKL family** | **Paralog used** | **Consensus sequence (Human, *Xenopus*, Zebrafish and Fugu*)** | **% seq. id.** | **# Ψ** |
| --- | --- | --- | --- | --- |
| LBX | LBX1 | RRKSRTAFTNHQIYELEKRFLYQKYLSPADRDQIAQQLGLTNAQVITWFQNRRAKLKRDL | 100 | 0 |
| NK2.1 | NKX2.1 | RRKRRVLFSQAQVYELERRFKQQKYLSAPEREHLASMIHLTPTQVKIWFQNHRYKMKRQA | 100 | 0 |
| NK3 | NKX3.2 | KKRSRAAFSHAQVFELERRFNHQRYLSGPERADLAASLKLTETQVKIWFQNRRYKTKRRQ | 100 | 0 |
| BARHL | BARHL2 | PRKARTAF-DHQLNQLERSFERQKYLSVQDRMDLAAALNLTDTQVKTWYQNRRTKWKRQT | 98,3 | 0 |
| BSX | BSX | RRKARTVFSDSQLSGLEKRFE-QRYLSTPERVELATALSLSETQVKTWFQNRRMKHKKQL | 98,3 | 0 |
| EMX | EMX1 (£) | PKRIRTAFSPSQLLRLERAFEKNHYVVGAERKQLA-SLSLSETQVKVWFQNRRTKYKRQK | 98,3 | 0 |
| HLX | HLX | RSWSRAVFSNLQRKGLEKRFE-QKYVTKPDRKQLAAMLGLTDAQVKVWFQNRRMKWRHSK | 98,3 | 0 |
| MSX | MSX1 | NRKPRTPFTT-QLLALERKFRQKQYLSIAERAEFSSSL-LTETQVKIWFQNRRAKAKRLQ | 96,7 | 1 |
| VAX | VAX2 | PKRTRTSFTAEQLYRLE-EFQRCQYVVGRERTELARQLNLSETQVKVWFQNRRTKQKKD- | 96,7 | 0 |
| BARX | BARX2 | PRRSRTIFTE-QL-GLEKKFQKQKYLSTPDRLDLAQSLGLTQLQVKTWYQNRRMKWKK-V | 95 | 0 |
| HHEX | HHEX | RKGGQVRFSNDQT-ELEK-FETQKYLSPPERKRLAK-LQLSERQVKTWFQNRRAKWRRLK | 95 | 0 |
| NK5 | HMX1 | KKKTRTVFSRSQVFQLESTFD-KRYLSS-ERAGLAA-L-LTETQVKIWFQNRRNKWKRQL | 93,3 | 0 |
| NK6 | NKX6.3 | KKHTRPTF-GHQIF-LEKTFEQTKYLAGPERARLA-SLGM-ESQVKVWFQNRRTKWRKKS | 93,3 | 0 |
| EN | EN2 | DKRPRTAFTA-QLQRLK-EFQTNRYLTEQRRQ-LAQEL-LNESQIKIWFQNKRAKIKKA- | 91,7 | 0 |
| DLX | DLX4 | -RKPRTIYSSLQLQ-L-QRFQ-TQYLALPERA-LAA-LGLTQTQVKIWFQNKRSKYKK-- | 88,3 | 0 |
| NK1 | NKX1.2 | PRRARTAFTYEQLVALE--FR--RYLSVCERL-LAL-L-LTETQVKIWFQNRRTKWKKQ- | 88,3 | 0 |
| TLX | TLX2 | RKKPRTSFSR-Q--ELE-RF-RQKYLASAERA-LAKAL-M-D-QVKTWFQNRRTKWRRQT | 85 | 0 |
| NK2.2 | NKX2.8 | -KKRRVLFSKAQT-ELERRFRQQRYLS-PER-QLA--L-LTPTQVKIWFQNHRYK-KR-- | 83,3 | 0 |
| NK4 | NKX2.6 (£) | RR-PRVLFSQ-QV--LERRFKQQRYLSAPER--LA--L-LTS-QVKIWFQNRRYKCKRQ- | 81,7 | 0 |
| DBX | DBX2 | -ILRRAVFSE-QR--LE--F--QKYISK--R--LA--L-LKE-QVKIWFQNRRMKWRN-- | 70 | 0 |
| NOTO | NOTO | -KR-RT-F---QL--LEK-F--Q---VG--R--LA--L-L-E-QV-VWFQNRR-K--KQ- | 53,3 | 0 |
| **VENTX** | **VENTX2** | --R-RT-FT--Q---LE--F--H-YL---ER---A----L-E-Q--TWFQNRRMK-KR-- | **48,3** | **6** |
| **NANOG** | **NANOG (&)** | ----R--FS--Q---L---F--Q-Y--------L-----L-YKQVK-WFQN-RMK----- | **36,7** | **10** |

*: For some families a different set of species was used, see legend for details

# Ψ: Number of processed pseudogenes in the human genome
