# Supplementary material for: Ventx Factors Function as Nanog-Like Guardians of Developmental Potential in Xenopus
Source: PLoS One. 2012 May 14;7(5):e36855. doi: 10.1371/journal.pone.0036855 (PMC3351468; doi:10.1371/journal.pone.0036855)
Supplement: Table S2 — Mammalian Nanog and Xenopus ventxs share striking functional similarities. Mammalian Nanog (left) and Xenopus ventx1/2 (right) are “regulated by” (A and B), “regulate” (C) and “interact” (D) with homologous pathways, transcription factors, genes and proteins, respectively. Most of these factors are known to regulate pluripotency and/or cell commitment and differentiation in mammals (indicated by P/C), while their counterparts in frog are known to be involved in dorso/ventral patterning during embryogenesis (indicated by D/V). References 78–110 are listed as Supplemental References in Supporting Information. (TIF) [file pone.0036855.s006.tif]

**TABLE S2. Mammalian *Nanog* and *Xenopus* *ventxs* share striking functional similarities.**

| **mammalian Nanog** | | | ***Xenopus* ventx1/2** | | |
| --- | --- | --- | --- | --- | --- |
| **A. Regulated by (signalling pathways)** | | | | | |
| BMP4 | P/C | [[81](#_ENREF_4)] | BMP4 | D/V | [32,33,55,[82](#_ENREF_5)] |
| NODAL/ACTIVIN | P/C | [[83](#_ENREF_6),[84](#_ENREF_7)] | NODAL/ACTIVIN | D/V | [[85](#_ENREF_8),[86](#_ENREF_9)] |
| FGF | P/C | [[87](#_ENREF_10)] | FGF | - | [[88](#_ENREF_11),[89](#_ENREF_12)] |
| WNT | P/C | [[90](#_ENREF_13)] | WNT | - | [[88](#_ENREF_11),[91](#_ENREF_14),[92](#_ENREF_15)] |

| **mammalian *Nanog*** | | | | ***Xenopus* *ventx1/2*** | | |
| --- | --- | --- | --- | --- | --- | --- |
| **B. Regulated by (transcription factors)** | | | | | | |
| POU5F1 | P/C | | [[93](#_ENREF_16)] | oct25/oct91 | D/V | [44,[94](#_ENREF_17)] |
| SMAD1 | P/C | | [[81](#_ENREF_4),[95](#_ENREF_18)] | smad1 | D/V | [34,[96](#_ENREF_19),[97](#_ENREF_20)] |
| SOX2 | P/C | | [[93](#_ENREF_16)] | sox2 | D/V | [[98](#_ENREF_21)] |
| STAT3 | P/C | | [[99](#_ENREF_22)] | stat3 | D/V | [[100](#_ENREF_23)] |
| TCF3 | P/C | | [[101](#_ENREF_24)] | tcf3 | D/V | [[102](#_ENREF_25)] |
| NANOG | | P/C | [[93](#_ENREF_16)] | Unknown | n.a. | n.a. |
| Unknown | | n.a. | n.a. | ventx1/2 | D/V | [33,34] |

| **mammalian Nanog** | | | | ***Xenopus* ventx1/2** | | |
| --- | --- | --- | --- | --- | --- | --- |
| **C. Regulates and/or binds to promoter region of by (transcription factors)** | | | | | | |
| *Bambi* | - | | [[103](#_ENREF_26)] | *bambi* | D/V | [[102](#_ENREF_25)] |
| *Bmp4* | P/C | | [[95](#_ENREF_18)] | *bmp4* | D/V | [32,33,[82](#_ENREF_5)] |
| *FoxA2* | | P/C | [[103](#_ENREF_26)] | *foxa2/ foxa4* | D/V | [42,[92](#_ENREF_15),[104](#_ENREF_27)] |
| *Gsc* | | P/C | [[83](#_ENREF_6),[103](#_ENREF_26)] | *gsc* | D/V | [32,33,34,37] |
| *Hesx1* | | P/C | [[103](#_ENREF_26)] | *xanf1* | - | [[105](#_ENREF_28)] |
| *Hhex* | | P/C | [[103](#_ENREF_26)] | *hhex* | - | [[92](#_ENREF_15),[106](#_ENREF_29)] |
| *Myf5* | | P/C | [[103](#_ENREF_26)] | *myf5* | - | [34,54] |
| *Nodal* | | P/C | [[83](#_ENREF_6),[95](#_ENREF_18),[103](#_ENREF_26)] | *xnr1* | D/V | [[104](#_ENREF_27)] |
| *Gata2* | | P/C | [[95](#_ENREF_18)] | *gata2* | D/V | [[107](#_ENREF_30),[108](#_ENREF_31),[109](#_ENREF_32)] |
| *Nanog* | | P/C | [[83](#_ENREF_6),[95](#_ENREF_18),[103](#_ENREF_26)] | n.a. | n.a. | n.a. |
| Unknown | | n.a. | n.a. | *ventx1/2* | D/V | [33,34,[96](#_ENREF_19)] |

| **mammalian Nanog** | | | | ***Xenopus* Ventx1/2** | | |
| --- | --- | --- | --- | --- | --- | --- |
| **D. Interacts with** | | | | | | |
| POU5F1 | P/C | [[110](#_ENREF_33)] | pou5f1.1 | | D/V | [44] |
| SMAD1 | P/C | [[99](#_ENREF_22)] | smad1 | | D/V | [[96](#_ENREF_19)] |
