# Supplementary material for: Ventx Factors Function as Nanog-Like Guardians of Developmental Potential in Xenopus
Source: PLoS One. 2012 May 14;7(5):e36855. doi: 10.1371/journal.pone.0036855 (PMC3351468; doi:10.1371/journal.pone.0036855)
Supplement: Table S3 — Primer pairs used for RT-QPCR experiments in this study. For each primer pair, the forward and reverse sequences are listed, as well as the original publications (references 111–125 are listed as Supplemental References in Supporting Information). (TIF) [file pone.0036855.s007.tif]

**TABLE S3: Primer pairs used for RT-QPCR experiments in this study.**

| **Gene** | **Original reference** | **Forward primer sequence** | **Reverse primer sequence** |
| --- | --- | --- | --- |
| ***bmp4*** | [[111](#_ENREF_34)] | 5′-AAG AGG ATG AGC TGC ACG AT-3′ | 5′-GCT GCT GAG GTT GAA CAC AA-3′ |
| ***eef1a1*** | [[112](#_ENREF_35)] | 5'-TGG ATA GCC CCT GTG TTG GAT T-3' | 5'-TCC ACG CAC ATT GGC TTT CCT-3' |
| ***eomes*** | [[113](#_ENREF_36)] | 5’-TGG TCC TCA AGG TCA AGT CC-3’ | 5’-GGG GAG TTT TCA TTG CTT GA-3’ |
| ***foxi1a*** | [[114](#_ENREF_37)] | 5’-CCA GAA CTG AAA TCT TAG CAA-3’ | 5’-TAA CAA AGA TAA AGC CAG AGG T-3’ |
| ***gsc*** | [[115](#_ENREF_38)] | 5’-TTC ACC GAT GAA CAA CTG GA-3’ | 5’-TTC CAC TTT TGG GCA TTT TC-3’ |
| ***hhex*** | [[116](#_ENREF_39)] | 5’-AAC AGC GCA TCT AAT GGG AC-3’ | 5’-CCT TTC CGC TTG TGC AGA GG-3’ |
| ***k81a1*** | XMMR | 5'-CAC CAG AAC ACA GAG TAC-3' | 5'-CAA CCT TCC CAT CAA CCA-3' |
| ***lim5*** | [[117](#_ENREF_40)] | 5’-GTG CAA CCT GAC CGA AAA AT-3’ | 5’-CTC GCT TTC CTG ACC AAG TC-3’ |
| ***mix1*** | This paper | 5’-GAA GGA CTT CCA GCA GCA TC-3’ | 5’-GCG TCT TTG GGT CTG ACA TT-3’ |
| ***mixer*** | [29] | 5’-CCT AGG ATG GAC ACG TTC AGC-3’ | 5’-GTG GAT CAG TGA AGC CCA CTG-3’ |
| ***myf5*** | [[118](#_ENREF_41)] | 5’-TAG CTG TTC AGA TGG CAT GTC T-3’ | 5’-CGG AAG GGA GTC AGT GCT AC-3’ |
| ***not*** | [29] | 5’-CTG CAT TTG GCC ACC ACC TGGC-3’ | 5’-GAT GAG CCA CAC GGG TGG GTA-3’ |
| ***oct91*** | [25] | 5’-TAG TGA TGG GCT GAG CAG TG-3’ | 5’-GGT GGT CTG GCT GAA TGT TT-3’ |
| ***odc*** | [[119](#_ENREF_42)] | 5’-TGA AAA CAT GGG TGC CTA CA-3’ | 5’-AAG TTC CAT TCC GCT CTC CT-3’ |
| ***siamois*** | [[120](#_ENREF_43)] | 5’-CTG TCC TAC AAG AGA CTC TG-3’ | 5’-TGT TGA CTG CAG ACT GTT GA-3’ |
| ***sox2*** | [[116](#_ENREF_39)] | 5’-CCA GTC CAC CTG TAG TCA CCT CT-3’ | 5’-CAC TTC TGC CCC AGG TAG GTA C-3’ |
| ***sox17*** | [[121](#_ENREF_44)] | 5'-GCA AGA TGC TTG GCA AGT CG-3' | 5'-GCT GAA GTT CTC TAG ACA CA-3' |
| ***tbx6*** | [[122](#_ENREF_45)] | 5’-CAG CCA ATC AGG AAC AAG G-3’ | 5’-GTT CTG TGC TGC ATC TGT GG-3’ |
| ***tfap2a*** | [[123](#_ENREF_46)] | 5’-TCC CAA CAG CCA TAC AGAC A-3’ | 5’-AGT TGG TGG CTG CAG AAA GT-3’ |
| ***t/bra*** | [[124](#_ENREF_47)] | 5’-TTC TGA AGG TGA GCA TGT CG-3’ | 5’-GTT TGA CTT TGC TAA AAG AGA CAG G-3’ |
| ***xnr5*** | [[113](#_ENREF_36)] | 5′-ATG AGG CCT CTG TCA ATG CT-3′ | 5′-GCC CTG AAT GTC TTG CAT CT-3′ |
| ***ventx2.1-b*** | [[125](#_ENREF_48)] | 5'-TTT CAG ATG CTC TAC CTG C-3' | 5'-CAA ATG GCC TTT CTT CCT G-3' |
| ***wnt8*** | [29] | 5’-GAT GTG ATG ACT CCA GAA ATGG C-3’ | 5’-CGA GAT CCG CTC ACC AAA TT-3’ |
