# Supplementary material for: Ventx Factors Function as Nanog-Like Guardians of Developmental Potential in Xenopus
Source: PLoS One. 2012 May 14;7(5):e36855. doi: 10.1371/journal.pone.0036855 (PMC3351468; doi:10.1371/journal.pone.0036855)
Supplement: Supporting Information S1 — The Supporting Information file contains Extended Experimental Procedures and Supplemental References. (DOC) [file pone.0036855.s008.doc]

**SUPPORTING INFORMATION**

**Extended Experimental Procedures**

**Reverse Transcriptase - PCR screening**

Total RNAs were extracted from *Xenopus laevis* ovaries, unfertilized and fertilized eggs (NF1), blastulae (NF8) and early gastrulae (NF10.5) using the RNeasy mini Kit (Qiagen), then reverse transcribed using superscript II reverse transcriptase (Invitrogen). The resulting cDNAs were screened by PCR using degenerate primers (MWG-Biotech). Primer design was based on the strict consensus sequence extracted from the *Nanog* homeoboxes found in amniotes (forward: 5’-TTYCARNNNCARAARTAYYTNWSNCC-3’ or 5’-TTYGTNNNNCARAARTAYYTNWSNCC-3’), in teleosts (forward: 5’-CNGCNTTYWSNGARWSNCARATG-3’ or 5’- CNGCNTTYWSNGARGARCARATG-3’, reverse: 5’-ACYTGYTTRTANGTNARNCCNG-3’) or in all vertebrates (reverse: 5’-TTYTGRAACCANGTYTTNAC-3’); deoxyinosine was used to reduce degeneracy. PCR reactions (94°C, 2’; 40x[94°C, 30’’; 45-55°C, 1’; 72°C, 30’’]; 72°C, 2’, with hot-start and annealing temperature gradient) were done in presence of ExTaq (Takara) on a MyCycler thermocycler (Biorad). No significant amplification was detected upon gel electrophoresis in presence of SybrSafe DNA Gel Stain fluorescent dye (Invitrogen). Additional PCR conditions did not yield amplification products either (primer concentrations from 0.2 to 1.0 µM, Mg2+ concentrations from 1 to 4 mM, alternate cycling conditions: touch-down, bottom-up). Specific primers for *ventx2.1-b* (also known as *xom*) and *ventx1.2* (also known as *vent-1*) were used as positive controls (see **Table S2**), leading to single band amplificons of the expected size in all experiments.

**Sequences retrieval**

We retrieved homeodomain sequences of all referenced NKL factors from *Homo sapiens*, *Gallus gallus,* *Xenopus tropicalis*, *Danio rerio*, *Branchiostoma floridae* and *Drosophila melanogaster* (referenced on the homeoDB2 website (http://homeodb.cbi.pku.edu.cn/)), as well as those from *Takifugu rubripes*, *Anolis carolinensis*, *Ornithorhynchus anatinus* and *Monodelphis domestica* (referenced on the ncbi, ensembl or Joint Genome Institute websites: http://www.ncbi.nlm.gov/, http://www.ensembl.org/, http://www.jgi.doe.gov/). When a given paralog was unknown in one of these species but present in a closely related one, the relevant sequences were retained (*Gasterosteus aculeatus* for *Danio rerio*, *Tetraodon nigroviridis* for *Takifugu rubripes*, *Taeniopygia guttata* for *Gallus gallus* and *Xenopus laevis* or *Ambystoma mexicanum* for *Xenopus tropicalis*). All these sequences were compiled and aligned using the Seaview software . This dataset as well as others used in this study are available upon request to the corresponding authors.

**Phylogenetic analyses**

Molecular phylogenetic analyses were performed on the 60 amino acids of the aligned NKL homeodomains using Maximum likelihood (JTT model of amino-acids substitution) as implemented in the PHYML software . Branch support was assessed using bootstrap replication (100 replicates).

**Conservation analysis**

For each NKL family conserved among vertebrates (Lbx, NK2.1, NK3, Bsx, Emx, Hlx, Barx, Msx, Vax, Hhex, NK5, NK6, En, Dlx, NK1, Tlx, Nk2.2, Dbx, Noto, Ventx and Nanog), the 60 amino acids of the homeodomains of *Homo sapiens*, *Xenopus tropicalis*, *Danio rerio* and *Takifugu rubripes* representatives were separately aligned. In order to always compare two tetrapod and two teleost sequences, when a given paralog was unknown in one of these species but present in a closely related one, the relevant sequences were retained (e.g. for Nanog, the *Ambystoma mexicanum* sequence was used instead of *Xenopus tropicalis*). For each set of orthologs, strict consensus sequences were obtained and the percentage of sequence identity computed using the Seaview software . In the case of families represented by multiple paralogs, only the least conserved subfamily was retained to generate **Table S1**. The number of processed pseudogenes found in the human genome for each NKL family was obtained from the homeoDB2 website .

**Supplemental References**

78. Zhong YF, Butts T, Holland PW (2008) HomeoDB: a database of homeobox gene diversity. Evol Dev 10: 516-518.

79. Gouy M, Guindon S, Gascuel O (2010) SeaView version 4: A multiplatform graphical user interface for sequence alignment and phylogenetic tree building. Mol Biol Evol 27: 221-224.

80. Guindon S, Gascuel O (2003) A simple, fast, and accurate algorithm to estimate large phylogenies by maximum likelihood. Syst Biol 52: 696-704.

81. Xu RH, Sampsell-Barron TL, Gu F, Root S, Peck RM, et al. (2008) NANOG is a direct target of TGFbeta/activin-mediated SMAD signaling in human ESCs. Cell Stem Cell 3: 196-206.

82. Schuler-Metz A, Knochel S, Kaufmann E, Knochel W (2000) The homeodomain transcription factor Xvent-2 mediates autocatalytic regulation of BMP-4 expression in Xenopus embryos. J Biol Chem 275: 34365-34374.

83. Vallier L, Mendjan S, Brown S, Chng Z, Teo A, et al. (2009) Activin/Nodal signalling maintains pluripotency by controlling Nanog expression. Development 136: 1339-1349.

84. Vallier L, Touboul T, Brown S, Cho C, Bilican B, et al. (2009) Signaling pathways controlling pluripotency and early cell fate decisions of human induced pluripotent stem cells. Stem Cells 27: 2655-2666.

85. Piepenburg O, Grimmer D, Williams PH, Smith JC (2004) Activin redux: specification of mesodermal pattern in Xenopus by graded concentrations of endogenous activin B. Development 131: 4977-4986.

86. Wessely O, Kim JI, Geissert D, Tran U, De Robertis EM (2004) Analysis of Spemann organizer formation in Xenopus embryos by cDNA macroarrays. Dev Biol 269: 552-566.

87. Nichols J, Silva J, Roode M, Smith A (2009) Suppression of Erk signalling promotes ground state pluripotency in the mouse embryo. Development 136: 3215-3222.

88. Ladher R, Mohun TJ, Smith JC, Snape AM (1996) Xom: a Xenopus homeobox gene that mediates the early effects of BMP-4. Development 122: 2385-2394.

89. Keren A, Keren-Politansky A, Bengal E (2008) A p38 MAPK-CREB pathway functions to pattern mesoderm in Xenopus. Dev Biol 322: 86-94.

90. Sumi T, Tsuneyoshi N, Nakatsuji N, Suemori H (2008) Defining early lineage specification of human embryonic stem cells by the orchestrated balance of canonical Wnt/beta-catenin, Activin/Nodal and BMP signaling. Development 135: 2969-2979.

91. Marom K, Levy V, Pillemer G, Fainsod A (2005) Temporal analysis of the early BMP functions identifies distinct anti-organizer and mesoderm patterning phases. Dev Biol 282: 442-454.

92. McLin VA, Rankin SA, Zorn AM (2007) Repression of Wnt/beta-catenin signaling in the anterior endoderm is essential for liver and pancreas development. Development 134: 2207-2217.

93. Boer B, Kopp J, Mallanna S, Desler M, Chakravarthy H, et al. (2007) Elevating the levels of Sox2 in embryonal carcinoma cells and embryonic stem cells inhibits the expression of Sox2:Oct-3/4 target genes. Nucleic Acids Res 35: 1773-1786.

94. Snir M, Ofir R, Elias S, Frank D (2006) Xenopus laevis POU91 protein, an Oct3/4 homologue, regulates competence transitions from mesoderm to neural cell fates. EMBO J 25: 3664-3674.

95. Loh YH, Wu Q, Chew JL, Vega VB, Zhang W, et al. (2006) The Oct4 and Nanog transcription network regulates pluripotency in mouse embryonic stem cells. Nat Genet 38: 431-440.

96. Henningfeld KA, Friedle H, Rastegar S, Knochel W (2002) Autoregulation of Xvent-2B; direct interaction and functional cooperation of Xvent-2 and Smad1. J Biol Chem 277: 2097-2103.

97. Lee HS, Park MJ, Lee SY, Hwang YS, Lee H, et al. (2002) Transcriptional regulation of Xbr-1a/Xvent-2 homeobox gene: analysis of its promoter region. Biochem Biophys Res Commun 298: 815-823.

98. Rogers CD, Harafuji N, Archer T, Cunningham DD, Casey ES (2009) Xenopus Sox3 activates sox2 and geminin and indirectly represses Xvent2 expression to induce neural progenitor formation at the expense of non-neural ectodermal derivatives. Mech Dev 126: 42-55.

99. Suzuki A, Raya A, Kawakami Y, Morita M, Matsui T, et al. (2006) Nanog binds to Smad1 and blocks bone morphogenetic protein-induced differentiation of embryonic stem cells. Proc Natl Acad Sci U S A 103: 10294-10299.

100. Nishinakamura R, Matsumoto Y, Matsuda T, Ariizumi T, Heike T, et al. (1999) Activation of Stat3 by cytokine receptor gp130 ventralizes Xenopus embryos independent of BMP-4. Dev Biol 216: 481-490.

101. Pereira L, Yi F, Merrill BJ (2006) Repression of Nanog gene transcription by Tcf3 limits embryonic stem cell self-renewal. Mol Cell Biol 26: 7479-7491.

102. Karaulanov E, Knochel W, Niehrs C (2004) Transcriptional regulation of BMP4 synexpression in transgenic Xenopus. EMBO J 23: 844-856.

103. Boyer LA, Lee TI, Cole MF, Johnstone SE, Levine SS, et al. (2005) Core transcriptional regulatory circuitry in human embryonic stem cells. Cell 122: 947-956.

104. Melby AE, Clements WK, Kimelman D (1999) Regulation of dorsal gene expression in Xenopus by the ventralizing homeodomain gene Vox. Dev Biol 211: 293-305.

105. Martynova N, Eroshkin F, Ermakova G, Bayramov A, Gray J, et al. (2004) Patterning the forebrain: FoxA4a/Pintallavis and Xvent2 determine the posterior limit of Xanf1 expression in the neural plate. Development 131: 2329-2338.

106. Rankin SA, Kormish J, Kofron M, Jegga A, Zorn AM (2011) A gene regulatory network controlling hhex transcription in the anterior endoderm of the organizer. Dev Biol 351: 297-310.

107. Hwang YS, Lee HS, Roh DH, Cha S, Lee SY, et al. (2003) Active repression of organizer genes by C-terminal domain of PV.1. Biochem Biophys Res Commun 308: 79-86.

108. Hwang YS, Seo JJ, Cha SW, Lee HS, Lee SY, et al. (2002) Antimorphic PV.1 causes secondary axis by inducing ectopic organizer. Biochem Biophys Res Commun 292: 1081-1086.

109. Xu RH, Ault KT, Kim J, Park MJ, Hwang YS, et al. (1999) Opposite effects of FGF and BMP-4 on embryonic blood formation: roles of PV.1 and GATA-2. Dev Biol 208: 352-361.

110. Liang J, Wan M, Zhang Y, Gu P, Xin H, et al. (2008) Nanog and Oct4 associate with unique transcriptional repression complexes in embryonic stem cells. Nat Cell Biol 10: 731-739.

111. Nichane M, de Croze N, Ren X, Souopgui J, Monsoro-Burq AH, et al. (2008) Hairy2-Id3 interactions play an essential role in Xenopus neural crest progenitor specification. Dev Biol 322: 355-367.

112. Fini JB, Pallud-Mothre S, Le Mevel S, Palmier K, Havens CM, et al. (2009) An innovative continuous flow system for monitoring heavy metal pollution in water using transgenic Xenopus laevis tadpoles. Environ Sci Technol 43: 8895-8900.

113. Kofron M, Wylie C, Heasman J (2004) The role of Mixer in patterning the early Xenopus embryo. Development 131: 2431-2441.

114. Matsuo-Takasaki M, Matsumura M, Sasai Y (2005) An essential role of Xenopus Foxi1a for ventral specification of the cephalic ectoderm during gastrulation. Development 132: 3885-3894.

115. Xanthos JB, Kofron M, Tao Q, Schaible K, Wylie C, et al. (2002) The roles of three signaling pathways in the formation and function of the Spemann Organizer. Development 129: 4027-4043.

116. Chang C, Hemmati-Brivanlou A (2000) A post-mid-blastula transition requirement for TGFbeta signaling in early endodermal specification. Mech Dev 90: 227-235.

117. Lim JW, Hummert P, Mills JC, Kroll KL (2011) Geminin cooperates with Polycomb to restrain multi-lineage commitment in the early embryo. Development 138: 33-44.

118. Vivien C, Scerbo P, Girardot F, Le Blay K, Demeneix BA, et al. (2012) Non-viral expression of mouse Oct4, Sox2 and Klf4 factors efficiently reprograms tadpole muscle fibers in vivo. J Biol Chem.

119. Morvan Dubois G, Sebillot A, Kuiper GG, Verhoelst CH, Darras VM, et al. (2006) Deiodinase activity is present in Xenopus laevis during early embryogenesis. Endocrinology 147: 4941-4949.

120. Heasman J, Kofron M, Wylie C (2000) Beta-catenin signaling activity dissected in the early Xenopus embryo: a novel antisense approach. Dev Biol 222: 124-134.

121. Xanthos JB, Kofron M, Wylie C, Heasman J (2001) Maternal VegT is the initiator of a molecular network specifying endoderm in Xenopus laevis. Development 128: 167-180.

122. Shi J, Severson C, Yang J, Wedlich D, Klymkowsky MW (2011) Snail2 controls mesodermal BMP/Wnt induction of neural crest. Development 138: 3135-3145.

123. Morgan MJ, Woltering JM, In der Rieden PM, Durston AJ, Thiery JP (2004) YY1 regulates the neural crest-associated slug gene in Xenopus laevis. J Biol Chem 279: 46826-46834.

124. Sun BI, Bush SM, Collins-Racie LA, LaVallie ER, DiBlasio-Smith EA, et al. (1999) derriere: a TGF-beta family member required for posterior development in Xenopus. Development 126: 1467-1482.

125. Gao H, Wu B, Giese R, Zhu Z (2007) Xom interacts with and stimulates transcriptional activity of LEF1/TCFs: implications for ventral cell fate determination during vertebrate embryogenesis. Cell Res 17: 345-356.
